# Burden of orofacial clefts from 1990–2021 at global, regional, and national levels

**DOI:** 10.3389/fped.2025.1502877

**Published:** 2025-03-21

**Authors:** Qinqin Ma, Jie Wei, Bo Peng, Jianying Liu, Shuixue Mo

**Affiliations:** ^1^ Department of Orthodontics, College of Stomatology, Guangxi Medical University, Nanning, Guangxi, China; ^2^ Guangxi Clinical Research Center for Craniofacial Deformity, Nanning, Guangxi, China; ^3^ Division of Hepatobiliary Surgery, The First Affiliated Hospital of Guangxi Medical University, Nanning, Guangxi, China; ^4^ Key Laboratory of Basic and Clinical Application Research for Hepatobiliary Diseases of Guangxi, Nanning, Guangxi, China; ^5^ Guangxi Key Laboratory of Immunology and Metabolism for Liver Diseases, Nanning, Guangxi, China

**Keywords:** orofacial clefts, global burden of disease, prevalence, mortality, disability-adjusted life years

## Abstract

**Objectives:**

We aimed to study and comprehensively evaluate the burden of OFCs at global, regional, and national levels.

**Methods:**

Based on data from the Global Burden of Disease database for 2021, we analysed the prevalence, mortality, and disability-adjusted life years (DALYs) of orofacial clefts (OFCs) from 1990–2021, categorised by sex, regions, and sociodemographic index (SDI). Numbers and age-standardised rates (ASRs) of the aforementioned indices were estimated through a systematic analysis of modelled data from the GBD 2021 study. Finally, the relationship between SDI and the epidemiological parameters of OFCs was evaluated.

**Results:**

In 2021, the global prevalence of OFCs included 4,124,007 cases, resulting in 1,719 deaths and 408,775 DALYs. From 1990–2021, prevalence cases decreased by 40.38%, while mortality rates, and DALYs decreased by 86.08%, and 68.33%, respectively. Moreover, the ASRs for prevalence, mortality, and DALYs demonstrated a decreasing trend during the period. In 2021, the highest age-standardised prevalence rates (ASPRs) of OFCs were recorded in South Asia, North Africa, the Middle East, and Central Asia. Nationally, Palestine, Qatar, and Bangladesh reported the three highest ASPRs in 2021. A nonlinear association was observed between the ASRs of OFCs and the SDI at regional and national levels.

**Conclusions:**

The global burden of OFCs decreased from 1990–2021. However, there is a disparity in disease burden across different regions, over 80% of the burden is borne by patients in low- and middle-income countries, the burden of OFCs remains a major public health challenge globally. Our findings will help to formulate appropriate policies to reduce the OFCs burden.

## 1 Background

Orofacial clefts (OFCs) are most common facial birth defects in the world (1). There are two types of OFCs; a majority of them are simple cleft lips, cleft palates, or facial clefts without any additional birth defects, termed the nonsyndromic type. The other category is the syndromic type, which is frequently associated with other birth defects,account for 15%–30% of cases (2). OFCs severely affect the patient's diet and, speech, facial, dental and other developmental issues; despite advances in medical knowledge and increasingly updated methods of effective prevention and treatment, OFCs remain a serious public health problem that imposes a significant burden on patients and society (3, 4). Furthermore, OFCs are associated with an elevated risk of death caused by congenital defects (5).

The aetiology of OFCs remains unclear, although genetic and environmental factors are currently considered the main causes (6). Recent genome-wide association studies have demonstrated that many genes and variants are associated with OFCs (7, 8). Environmental factors, including prominent drug use, alcohol consumption, smoking, parental age, and environmental toxins, may also influence the development of OFCs (9, 10). Therefore, supplementation of folic acid before pregnancy and during pregnancy, smoking cessation, alcohol withdrawal, avoidance of teratogenic drugs, reduction of air pollution, prohibition of recent marriage, prenatal genetic testing, genetic counseling, etc., can reduce the incidence of OFC (9, 10).

Epidemiological studies reveal significant geographic, ethnic disparities. Global data have shown that the overall incidence of OFCs in the world is one in 700 live births. Another recent systematic review revealed that in low- and middle-income countries, approximately one in 730 children was born with OFCs (11). Furthermore, the birth prevalence of OFCs worldwide has been reported to vary significantly. A previous systematic review showed that the OFC birth rate was higher in Asia than in other regions, at about 1.57 (1.54–1.60)/1,000 live births (12). However, these studies are only on the prevalence of OFC in regions or countries, and global data on all countries and regions is rare.To date, little global research has been done to assess the world's burden of OFCs across all countries and regions (13, 14). The previous studies focused on more developed countries or regions, and large gaps existed in registry data from low-income countries (13, 14). Further, there are no updated global studies on OFCs that provide comprehensive data on its epidemiology and burden. Moreover, the trends in prevalence, deaths, and DALYs of OFCs over time remain unexplored at these levels. Therefore, there is an urgent need to acquire precise data on OFCs worldwide and to allocate adequate resources for disease control and preventive purposes.

We analyzed data from the Global Burden of Disease Study (GBD) 2021 to obtain comprehensive information on the burden of OFCs, including prevalence, mortality, and DALYs by socio-demographic indices (SDIs), counts, and age-standardized rates (ASRs) at the global, regional, and national levels. This accurate information on the burden of OFCs can help to formulate appropriate policies to reduce the OFCs burden.

## 2 Methods

### 2.1 Overview and data source

Using the latest epidemiological data and enhanced standardized methodologies, the 2021 GBD study provides an in-depth assessment of health damage caused by 369 diseases, injuries and impairments and 88 risk factors in 204 countries and territories (15, 16). The Global Health Data Exchange query tool (https://ghdx.healthdata.org/gbd-2021/sources) provides detailed information on OFCs assessments. The data obtained for our study included prevalence, death, and DALY numbers of OFCs.

Prevalence reports the proportion of people in a population with a particular disease, and DisMod-MR21(Disease Modeling Meta-Regression) is a Bayesian disease modelling meta-regression tool can estimate prevalence rates across time, geography, age, and gender based on the data (16). In this research, DALYs quantify the burden of disease and are defined as the sum of years of life lost (YLLs) and years lived with disability (YLDs) for OFCs at each site, age group, sex, and point in time (15–17). YLLs were calculated by multiplying cause-age-sex-location-year-specific deaths by the standard life expectancy at the age that the death occurred. YLDs were calculated by multiplying the cause-age-sex-location-year-specific prevalence of sequelae with their corresponding disability weights for OFCs (16). The ASR denotes the number of prevalence cases, deaths, or DALYs per 100,000 population, adjusted for differences in population age (16). ASRs were calculated using the following formula:

$$\mathrm{ASRs}=\frac{\sum_{i=1}^{A}a_{i}w_{i}}{\sum_{i=1}^{A}w_{i}}\times 100,000$$

where *ai* represents the age-specific rate in the *i*^th^ age group,*w* represents the number of people (or the weight) in the same, *i*^th^ age group from among the selected reference standard population, and *A* represents the number of age groups.

The GBD study ensured the comparability of estimates across countries and over time by utilising age-standardised rates to accommodate variations in population age structures. The SDI is a composite measure comprising lag-distributed income per capita, average years of education for individuals aged 15 years or older, and fertility rates among females younger than 25 years (16). GBD 2021 divides the 204 countries and territories into five SDI-based zones: low, medium-low, medium, medium-high and high. This categorisation was applied to stratify regions or countries into five levels based on the SDI (16).

### 2.2 OFC definition

The GBD case definition OFCs include isolated cleft lip, isolated cleft palate, and combined cleft lip and palate, which corresponds to International Classification of Disease Version ICD-10 codes Q35.2–37.9 (16).

### 2.3 Statistical analysis

The relationship between ASR and SDI was assessed by Spearman's correlation coefficient. If the *p*-value of Pearson's correlation coefficient is less than 0.05, it indicates that there is a significant positive correlation between these two variables. All statistical data were generated using R software version4.3.3(https://www.r-project.org/) and data were visualised using “ggplot2” package.

## 3 Results

### 3.1 Global burden of orofacial clefts

**Figure 1:**
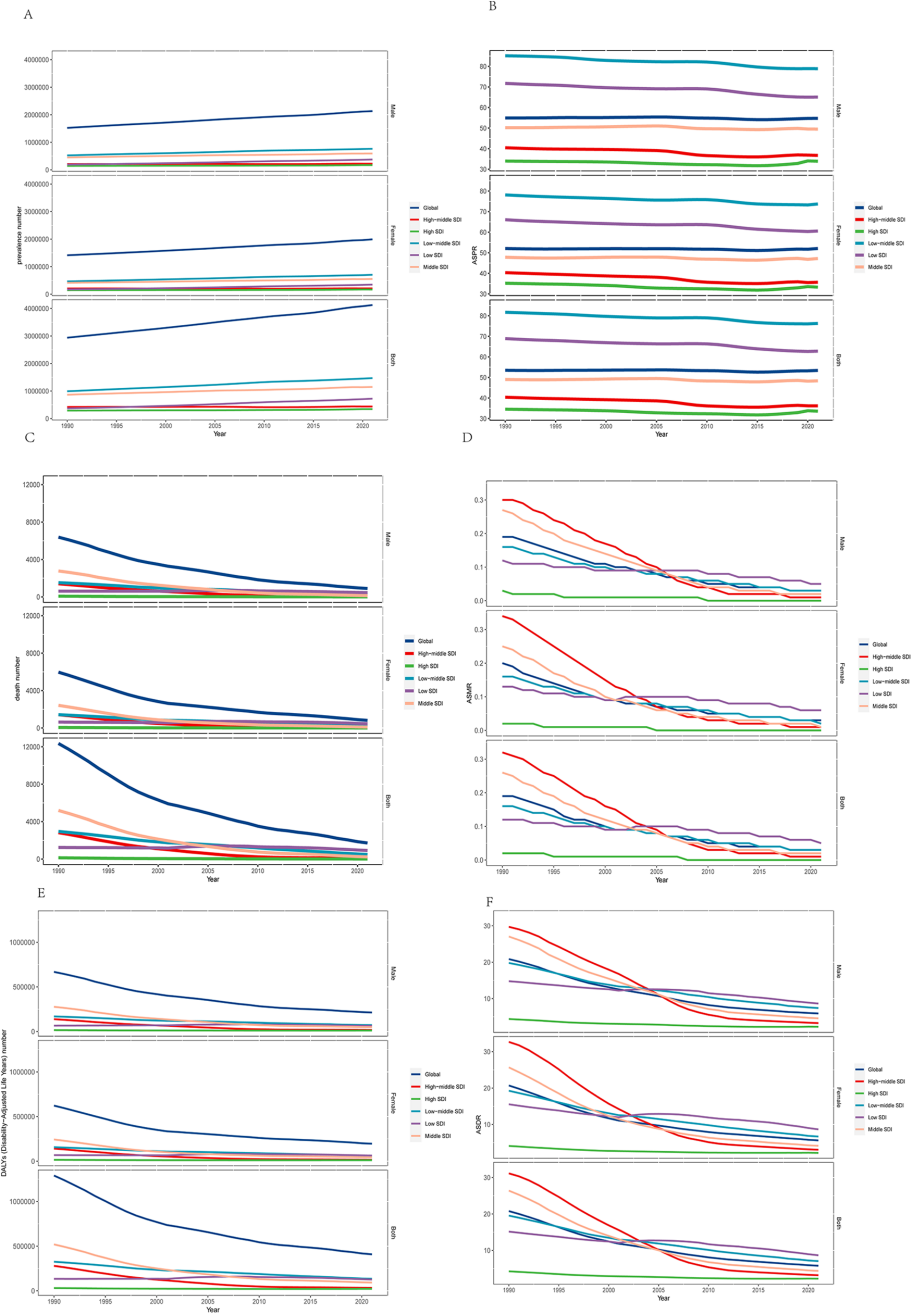
**(A)** The numbers of prevalence, **(B)** age-standardized prevalence rate, **(C)** numbers of deaths, **(D)** age-standardized death rate, **(E)** numbers of DALYs, and **(F)** numbers of DALYs are illustrated for orofacial clefts at the global and regional levels from 1990 through 2021. DALYs, Disability-Adjusted Life Years. SDI, Sociodemographic index.

In 2021, the global prevalence of OFC cases was 4,124,007 (95% UI: 3,318,693–5,026,200), with 81.0% of these patients residing in low and middle SDI countries (Table 1, Figure 1A). This represented a 40.68% increase from the 2,937,707 cases in 1990 (95% UI: 2,389,358–3,535,594) (Table 1). The global ASPR decreased from 53.47 per 100,000 populations in 1990 (95% UI: 43.44–64.48) to 53.42 per 100,000 populations in 2021 (95% UI: 42.02–65.03), with a percentage change of −0.001 (95% UI: −0.048–0.04) (Table 1, Figures 1B). The number of deaths due to OFCs was 1,719 in 2021 (95% UI: 485–5,437), which represented an 86.09% decrease from the 17,230 deaths in 1980 (95% UI: 5,892–35,094) (Table 1, Figures 1C,D). Notably, most deaths from this disease (96.1%) occurred in low- and middle-SDI countries (Table 1). The global ASMR decreased from 0.30 per 100,000 populations in 1980 (95% UI: 0.10–0.61) to 0.03 per 100,000 populations in 2021 (95% UI: 0.01–0.07), with a percentage change of −0.86 (−0.73–0.92). The estimated number of DALYs for OFCs was 1,290,533 in 1990 (95% UI: 590,789–2,246,407) and 408,775 in 2021 (95% UI: 252,320–671,120), with an ASDR of 20.78 in 1990 (9.71 –35.78) and 5.78 in 2021 (3.84–9.82) per 100,000 populations. This number decreased by 68.33% from 1990–2021 (Table 1 and Figures 1E, F).

**Table 1:** Prevalence, death cases, and disability-adjusted life years (DALYs) for orofacial clefts in 2021 and percentage change in age-standardized rates (ASRs) per 100,000 population from 1990–2021 by global burden of disease (GBD).

	Prevalence (95% uncertainty interval)			Death (95% uncertainty interval)			DALYs (95% uncertainty interval)		
Location name	Number 2021	ASR per 100,000 Population (95% UI) in 2021	Percentage change in ASRs per 100,000 population (95% UI)	Number 2021	ASR per 100,000 population (95% UI) in 2021	Percentage change in ASRs per 100,000 population (95% UI)	Number 2021	ASR per 100,000 population (95% UI) in 2021	Percentage change in ASRs per 100,000 population (95% UI)
Global	4,124,007 (3,318,692–5,026,200)	53.42 (43.02 –65.03)	−0.001 (−0.05 to 0.042)	1,719 (485–4,409)	0.03 (0.008–0.07)	−0.86 (−0.92 to −0.73)	408,775 (252,320–671,120)	5.78 (3.49–9.82)	−0.72 (−0.81 to −0.55)
Sex									
Male	2,132,361 (1,711,480–2,594,810)	54.76 (44.01 –66.54)	−0.003 (−0.05 to 0.037)	901 (209–2,788)	0.03 (0.006–0.09)	−0.86 (−0.92 to −0.69)	213,233 (124,472–384,612)	5.92 (3.34–11.14)	−0.72 (−0.83 to −0.46)
Female	1,991,646 (1,603,507–24,22,195	52.07 (42.05 –63.22)	0.0001 (−0.05 to 0.049)	818 (238–3,046)	0.03 (0.008–0.10)	−0.86 (−0.93 to −0.68)	195,542 (116,785–391,730)	5.63 (3.29–12.16)	−0.73 (−0.82 to −0.45)
High-middle SDI	192,022 (158,083–236,899)	36.20 （29.59–43.49）	−0.1 (−0.17 to −0.04)	57 (31–91)	0.01 (0.005–0.02)	−0.97 (−0.99 to −0.93)	32,496 (21,979–48,009)	3.17 (2.19–4.46)	−0.90 (−0.95 to −0.77)
High SDI	36,562 (30,755–43,330)	33.60 （27.03–40.57)	−0.03 (−0.09to 0.019)	7 (3–11)	0.001 (0.006–0.002	−0.94 (−0.97 to −0.87)	22,031 (13,636–33,497)	2.23 (1.41–3.36)	−0.48 (−0.68 to −0.25)
Low-middle SDI	336,498 (271,989–426,247)	76.29 (60.73–93.60)	−0.07 (−0.12 to −0.003)	497 (177–1,069)	0.03 (0.009–0.06)	−0.84 (−0.91 to −0.64)	134,880 (88,416–201,658)	7.05 (4.61–10.57)	−0.64 (−0.81 to −0.36)
Low SDI	139,675 (112,677–177,213)	62.80 （50.48–76.82）	−0.09 (−0.14 to −0.03)	917 (111–3,316)	0.05 (0.007–0.20)	−0.56 (−0.71 to −0.22)	126,685 (49,746–344,501)	8.65 (3.88–21.66)	−0.43 (−0.63 to −0.09)
Middle SDI	604,765 (500,791–731,047)	48.36 (39.24–58.82)	−0.01 (−0.07 to 0.041)	238 (142–360)	0.02 (0.009–0.02)	−0.94 (−0.97 to −0.87)	92,289 (65,014–129,542)	4.37 (3.11–6.10)	−0.83 (−0.90 to −0.69)
Australasia	8,317 (6,674–10,155)	28.58 (22.89–34.62)	−0.09 (−0.27 to - 0.13)	0.21 (0.03–0.45)	0.001 (0.0002 to 0.003)	−0.40 (−0.88 to 0.36)	551 (349–839)	1.93 (1.24–2.92)	−0.12 (−0.28 to 0.07)
Caribbean	21,561 (17,052–26,692)	46.19 (36.55–57.03)	0.50 (0.37 to 0.65)	8.11 (1.68–20.13)	0.02 (0.004 to 0.05)	−0.56 (−0.78 to −0.03)	2,049 (1,218–3,329)	4.75 (2.74–7.97)	−0.24 (−0.57 to 0.20)
Central Asia	76,835 (62,047–92,648)	79.97 (64.58–96.45)	−0.13 (−0.21 to −0.05)	9.60 (5.73–16.57)	0.01 (0.006 to 0.017)	−0.66 (−0.85to −0.30)	5,597 (3,769–8,352)	5.79 (3.90–8.64)	−0.30 (−0.47 to −0.14)
Central Europe	30,464 (24,477–36,896)	28.41 (23.05–34.16)	−0.19 (−0.25 to −0.12)	0.29 (0.11–0.59)	0.0006 (0.0002 to 0.001)	−0.97 (−0.99 to −0.95)	1,941 (1,199–2,954)	1.85 （1.16–2.76)	−0.56 (−0.70 to −0.44)
Central Latin America	96,757 (78,696–117,246)	39.11 (31.91–47.28)	−0.15 (−0.21 to −0.09)	41.17 (24.16–60.23)	0.02 (0.01 to 0.03)	−0.82 (−0.88 to −0.74)	9,695 (7,000–13,401)	4.38 (3.19–5.93)	−0.68 (−0.75to −0.57)
Central Sub-Saharan Africa	55,174 (44,009–68,507)	38.33 (30.59–47.80)	−0.03 (−0.14 to 0.10)	58.49 (7.21–239.72)	0.03 (0.003 to 0.11)	−0.60 (−0.78 to −0.09)	8,642 (3,614–24,279)	4.81 (2.22–12.11)	−0.44 (− 0.69 to 0.01)
East Asia	429,541 (347,840–519,201)	31.13 (25.49–637.10)	−0.19 (−0.26 to −0.11)	103.05 (52.07–169.71)	0.02 (0.009 to 0.03)	−0.97 (−0.98 to −0.92)	36,372 (24,863–52,967)	3.61 (2.44–5.17)	−0.93 (−0.96 to −0.83)
Eastern Europe	52,313 (41,814–64,113)	26.84 (21.47–32.65)	−0.16 (−0.22 to –0.10)	2.08 (0.83–3.55)	0.002 (0.0009 to 0.004)	−0.88 (−0.94 to −0.83)	3,538 (2,201–5,392)	1.93 (1.27–2.87)	−0.50 (−0.61 to −0.39)
Eastern Sub-Saharan Africa	221,976 (179,211–271,860)	49.66 (39.80–61.27)	−0.05 (−0.12 to 0.02)	256.95 (26.65–1,121.51)	0.04 (0.004 to 0.17)	−0.65 (−0.79 to −0.17)	36,757 (14,299–113,593)	6.58 (2.87–18.40)	−0.50 (−0.70 to −0.09)
Andean Latin America	33,534 (27,121–40,759)	51.08 (41.36–61.99)	0.20 (0.06 to 0.35)	12.62 (7.09–20.05)	0.021 (0.012 to 0.033)	−0.94 (−0.97 to −0.87)	2,418 (1,624–3,403)	5.05 (3.69–7.11)	−0.84 (−0.90 to −0.71)
High-income Asia Pacific	90,185 (71,751–11,736)	52.88 (42.26–64.61)	−0.05 (−0.17 to 0.03)	0.09 (0.04–0.15)	0.0002 (0.0002 to 0.0003)	−0.99 (−0.10 to −0.98)	5,517 (3,380–8,454)	3.29 (2.00–5.07)	−0.35 (−0.57 to −0.14)
High-income North America	68,252 (52,115–86,706)	19.74 (15.08–24.93	0.03 (−0.04 to 0.10)	0.68 (0.35–0.97)	0.0003 (0.0002 to 0.0005)	−0.95 (−0.97 to −0.92)	4,438 (2,676–6,903)	1.30 (0.79–2.01)	−0.28 (−0.41 to −0.16)
North Africa and Middle East	532,166 (425,775–646,874)	85.22 (68.24–103.52)	−0.05 (−0.12 to 0.02)	288.25 (95.27–1,051.88)	0.05 （0.02 to 0.18)	−0.83 (−0.92 to −0.65)	58,524 (34,890–125.072)	9.67 (5.72–21.17)	−0.70 (−0.81 to −0.43)
Oceania	4,786 (3,805–5,948)	33.38 (26.32–41.49)	0.3 (0.19 to 0.41)	35.08 (6.39–90.57)	0.17 (0.03 to 0.45)	−0.51 (−0.75 to −0.11)	3,446 (859–8,440)	17.52 (4.82–42.20)	−0.47 (−0.72 to 0.12)
South Asia	1,633,291 (1,295,458–2,022,506)	89.06 (70.71–110.10)	−0.11 (−0.17 to −0.05)	252.02 (41.72–862.22)	0.02 (0.003 to 0.06)	−0.87 (−0.94 to −0.67)	122,622 (76,631–193,338)	6.93 (4.24–11.04)	−0.61 (−0.81 to −022)
Southeast Asia	294,297 (238,650–359,900)	42.97 (34.87–52.37)	0.51 (0.39 to 0.62)	191.06 (82.75–363.24)	0.04 (0.02 to 0.07)	−0.79 (−0.88 to −0.52)	35,297 (23,249–53,116)	5.81 (3.73–9.00)	−0.65 (−0.78 to −0.32)
Southern Latin America	16,307 (12,730–19,985)	58.05 (47.05–71.84)	−0.06 (−0.20 to −0.08)	0.75 (0.32–1.41)	0.002 (0.001 to 0.004)	−0.75 (−0.90 to −0.46)	1,114 (712–1,712)	1.81 (1.20–2.74)	−0.26 (−0.45 to −0.09)
Southern Sub-Saharan Africa	46,725 (37,840–57,922)	58.05 (47.05–71.84)	−0.05 (−0.12 to 0.01)	36.18 (14.85–65.68)	0.05 (0.02 to 0.08)	−0.43 (−0.68 to −0.12)	6,098 (3,886–8,970)	7.68 (4.89–11.37)	−0.30 (−0.52 to 0.05)
Tropical Latin America	53,197 (43,518–63,293)	24.14 19.83–28.72)	−0.19 (−0.24 to 0.13)	17.91 (111.15–25.28)	0.01 (0.007 to 0.015)	−0.89 (−0.93 to −0.85)	4,985 (3,585–7,009)	2.50 (1.79–3.42)	−0.77 (−0.82 to −0.70)
Western Europe	106,620 (86,208–128,180)	26.56 (21.57–31.65)	−0.14 (−0.11 to −0.06)	1.28 (0.60–2.03)	0.0006 (0.0003to 0.001)	−0.95 (−0.97 to −0.92)	6,842 (4,201–10,342)	1.74 (1.09–2.59)	−0.43 (−0.54 to −0.32)
Western Sub-Saharan Africa	251,080 (202,129–306,334)	48.20 (38.53–59.25)	−0.07 (−0.13 to –0.01)	402.75 (45.68–1,900.36)	0.05 (0.005 to0.22)	−0.44 (−0.68 to −0.35)	51,539 (18,210- 184,528)	7.26 (3.02–23.27)	−0.33 (−0.61 to 0.10)

Data in parentheses represent the 95% uncertainty intervals. ’SDI’ stands for Socio-demographic Index.

### 3.2 Regional burden of orofacial clefts

**Figure 2:**
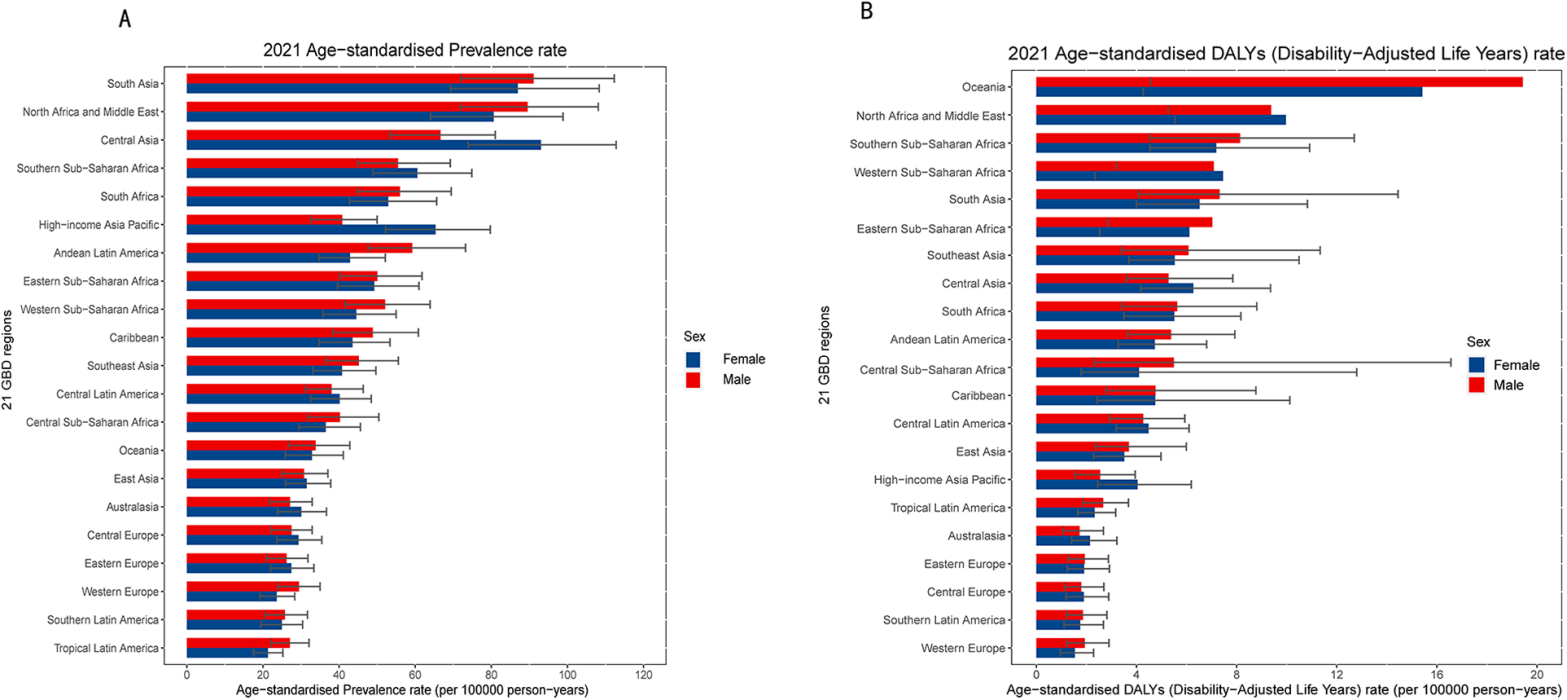
The age-standardized prevalence **(A)** and age-standardized DALYs (disability-adjusted life years) **(B)** rate of orofacial clefts in 2021 for 21 GBD region, by sex.

In 2021, studies analysed across regions showed that the ASPR of OFCs was the highest in regions with a low-middle SDI, reaching 76.29 per 100,000 population (95% UI: 60.73–93.60) (Table 1). At the regional level, the highest ASPRs of OFCs per 100,000 population in 2021 were observed in South Asia [89.06 (70.71–110.12)], North Africa and the Middle East [85.22 (68.24–103.54)], and Central Asia [79.97 (64.58–96.45)] (Table 1, Figure 2A). In contrast, the lowest ASPRs of OFCs per 100,000 population in 2021 were recorded in High-income North America [19.74 (15.08–24.93)], Tropical Latin America [24.14 (19.83–28.72)], and Southern Latin America [25.36 (20.0–31.05)] (Table 1, Figure 2A). The percentage change in ASPRs of OFCs from 1990–2021 showed a decrease in most regions (Table 1). The most significant increases were observed in Southeast Asia (50.53%, 95% CI: 39.08%–62.17%), followed by the Caribbean [50.39% (37.02%–65.45%)] and Oceania [29.64% (19.48%–40.51%)] from 1990–2021. Meanwhile, some regional trends indicated a decrease between 1990 and 2021, with the most substantial reductions recorded in Tropical Latin America [−18.7% (−24.1%–−12.64%)], East Asia [−18.56% (−26.34%–−10.87%)], and Central Europe [−18.54% (−25.48%–−11.59%)] (Table 1).

In addition, the number of prevalent cases varies among the GBD regions in 2021. South Asia [1,633,291 (1,295,458–2,022,506)], North Africa and Middle East [532,166 (425,775–646,873)], and East Asia [429,541 (347,840–519,201)] reported the highest number of prevalent cases in 2021 (Table 1 and Supplementarty Figure S1). The highest regional ASMR for OFCs was noted in Oceania [0.17 (0.03–0.44)] per 100,000, followed by North Africa and Middle East [0.05 (0.01–0.18)]. Conversely, no deaths from orofacial clefts were recorded in the High-income Asia Pacific, Australasia, Central Europe, High-income North America, or Southern Latin America. Between 1990 and 2021, the ASMR displayed a decreasing trend across all regions (Table 1 and Supplementary Figure S2). Furthermore, the highest number of death cases in 2021 was recorded in Western Sub-Saharan Africa [402 (47–1,990)], North Africa and the Middle East [288 (95–1,051)], and Eastern Sub-Saharan Africa [257 (27–1,122)] (Table 1 and Supplementary Figure S3).

In 2021, the highest ASDR of OFCs per 100,000 population was observed in Oceania [6.65 (4.1–9.91)], North Africa and the Middle East [5.63 (3.47–18.4)], and Southern Sub-Saharan Africa [3.29 (2.03–4.92)]. Conversely, the lowest ASDRs of OFCs in 2021 were recorded in High-income North America [0.96 (0.59–1.43)], Tropical Latin America [1.29 (0.80–1.89)], and Southern Latin America [1.51 (0.89–2.25)] (Table 1 and Figures 2B). From 1990 to 2021, the ASDR showed a decreasing trend across all regions, with the most significant declines observed in East Asia [−0.97 (−0.83–−0.96)], Andean Latin America [−0.84 (−0.31–−0.72)], and Tropical Latin America [−0.77 (−0.83–1.69)] (Table 1).

### 3.3 National burden of orofacial clefts

**Figure 3:**
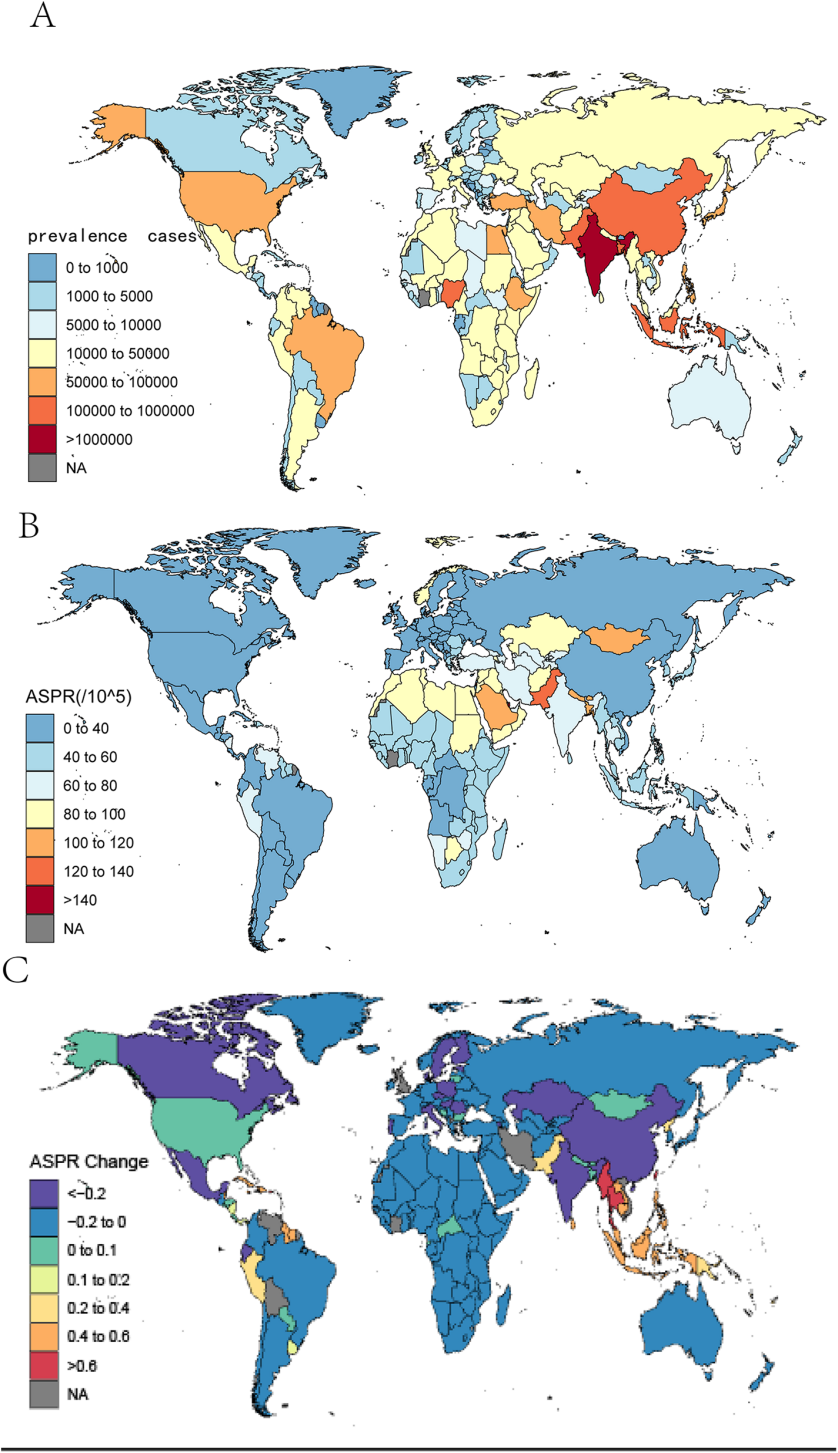
The global prevalence cases **(A)** and ASPR **(B)** of orofacial clefts per 100,000 population in 2021, and the relative change **(C)** in prevalence rate of orofacial clefts between 1990 and 2021, by country and territory. ASPR, age-standardized prevalence rate.

In 2021, the ASPR of OFCs varied from 9.07 to 147.15 cases per 100,000 populations at the national level. Countries with the highest ASPR included Palestine [147.15 (119.61–171.37)], Qatar [140.47 (111.24–173.15)], and Bangladesh [136.06 (107.62–169.11)]. Conversely, the countries with the lowest ASPR of OFCs were Canada [9.07 (7.15–11.05)], Greenland [13.72 (10.77–16.87)], and Spain [14.82 (11.85–18.03)] (Figure 3B). The percentage change in ASPR from 1990–2021 differed substantially among countries. Notably, the most significant increases were observed in Taiwan (Province of China) [0.76 (0.58–0.99)], Thailand [0.76 (0.52–1.04)], and Puerto Rico [0.66 (0.47–0.85)]. In contrast, the largest decreasing trends were noted in Finland [−34.51% (−41.51%–−25.97%)], Hungary [−32.13% (−45.42–−17.7%)], and Estonia [−30.33% (−37.74%–−21.62%)](Figure 3C). The top three countries with the highest prevalence of OFCs in 2021 were India [1,083,094 (860,196–1,350,729)], China [410,198 (331,815–495,792)], and Pakistan [326,252 (258,128–404,942)] (Figure 3A). At the national level, the number of deaths from OFCs in 2021 across the 204 countries ranged from 0–164.59 per 100,000 populations (Figure S5). The highest death rates occurred in India [164.59 (23.051–626.68)], Nigeria [489.03 (20.84–707.08)], and Afghanistan [109.68 (4.41–631.00)] (Supplementray Figure S6). From 1990–2021, the ASMR showed a decreasing trend in most countries, with a few exceptions. The largest decreases in the ASMR of OFCs occurred in the United Kingdom [−99.89% (−99.84– −99.85%)] and Croatia [−99.8% (−99.454%– −99.94%)], whereas the most significant increases during the same period were observed in Trinidad and Tobago [79.25% (−50%–419.27%)] and Afghanistan [38.83% (−54.6%–1128%)]. (Supplementray Figure S7).

At the national level, the ASDR of OFCs varied from 0.61–22.53 per 100,000 populations in 2021. Afghanistan, Lao People's Democratic Republic, and Papua New Guinea reported the highest ASDRs, with figures of 22.52 (5.8–103), 20.15 (8.62–41.91), and 19.76 (5.24–48.40), respectively. Conversely, the lowest ASDRs were recorded in Canada [0.61 (0.37–0.93)], Greenland [0.98 (0.60–1.49)], and Israel [1.04 (0.64–1.58)] (Supplementray Figure S8). Notably, Puerto Rico, Trinidad and Tobago, and Barbados observed the most significant increases in ASDR between 1990 and 2021, with changes of 63.13% (28.24%–99.52%), 56.73% (5%–124.33%), and 44.9% (16.01%–79.89%), respectively. In contrast, China, Iran (Islamic Republic of), and Turkey experienced the most substantial decreases, with reductions of −93% (−83.64%–96.47%), −92.02% (−78.65%– −95.95%), and −90.98% (−73.32– −95.32%) (Supplementray Figure S9). Furthermore, the highest number of DALYs from OFCs in 2021 was reported by India [80,917 (50,170–127,939)], China [34,629 (23,632–50,416)], and Pakistan [26,089 (15,328–45,250)] (Supplementray Figure S10).

### 3.4 Burden of orofacial clefts by SDI

**Figure 4:**
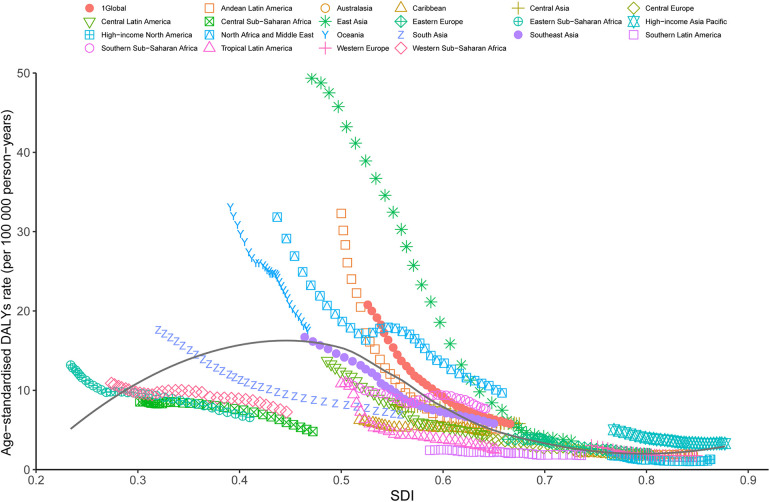
Trends in ASDR of orofacial clefts across 21 global burden of disease study regions are illustrated by sociodemographic index (SDI)for both sexes combined from 1990 to 2021. The black line indicates expected values. DALYs, disability-adjusted life years; ASDR, age standardized DALY rate.

A general negative association was observed between the ASDR of OFCs and the SDI globally and across all GBD regions. At the global level, the observed burden of OFCs exceeded expectations in regions with a lower SDI. Conversely, in regions with a higher SDI, the observed burden of OFCs dropped below the expected level. Regionally, from 1990–2021, the observed burden estimates for OFCs surpassed expected levels based on the SDI in East Asia, Andean Latin America, North Africa and Middle East, and Oceania. However, Southern Latin America, Tropical Latin America, Central Latin America, High-income North America, Caribbean, and Central Sub-Saharan Africa, exhibited a lower-than-expected burden of OFCs throughout the measurement periods (Figure 4). The association between prevalence, death, and SDI is described in the additional file (Supplementray Figures S11, S12).

**Figure 5:**
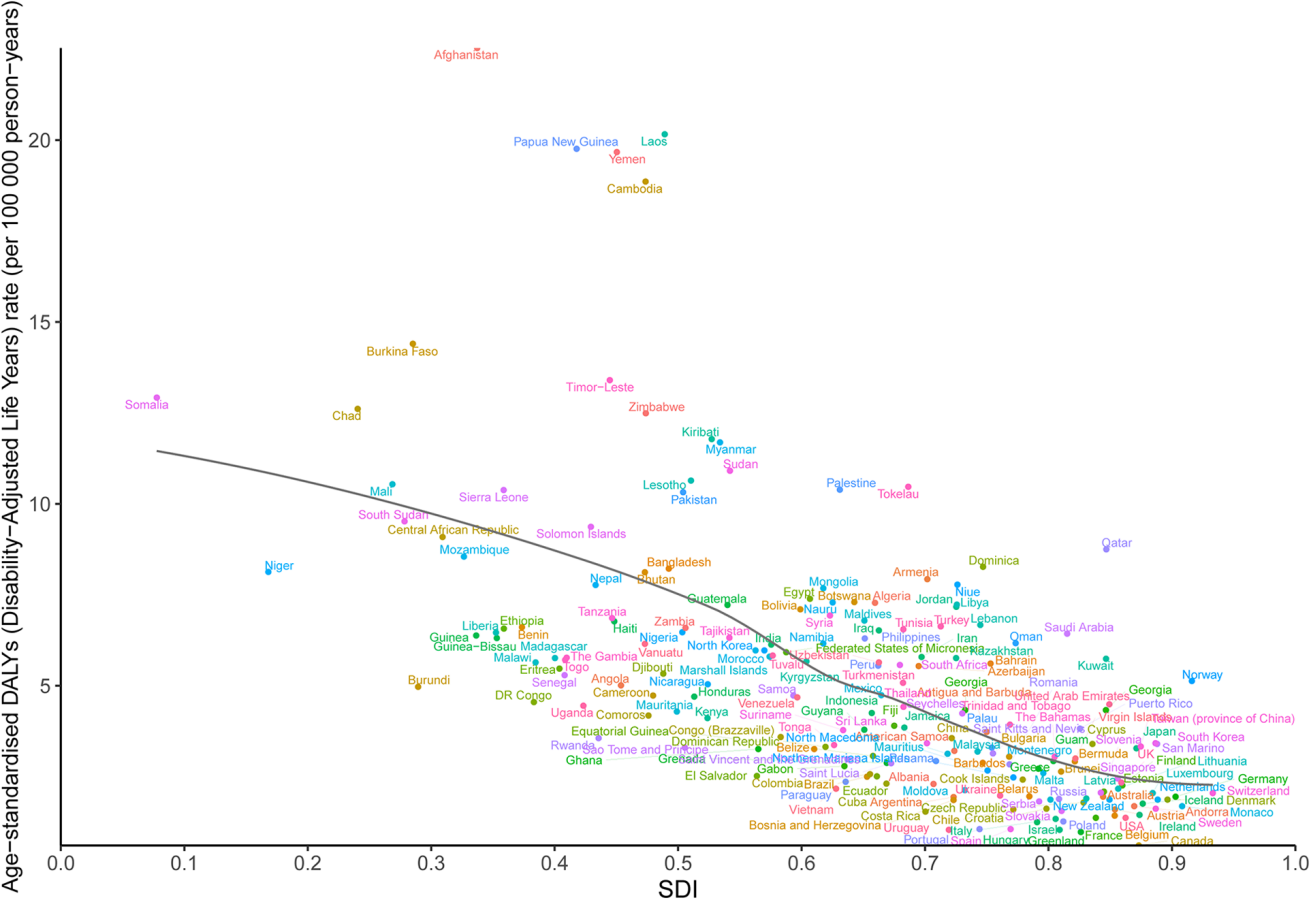
ASDR of orofacial clefts in 204 countries and territories and SDI in 2021. The black line indicates the expected values based on sociodemographic index and disease rates in all locations. DALYs, disability-adjusted life years; SDI, sociodemographic index; ASDR, agestandardized DALY rate.

In 2021, a negative correlation between ASDR and SDI of OFCs was also evident at the national level. Countries such as Afghanistan, Papua New Guinea, Laos, Yemen, Cambodia, and Burkina, among others, reported a significantly higher burden of OFCs than expected based on their SDI. In contrast, Canada, Greenland, Hungary, Portugal, and other countries or territories exhibited a much lower-than-expected ASDR (Figure 5). Similarly, negative associations were noted between the SDI and ASPR, ASMR of OFCs in 2021. (Supplementray Figures S13, S14).

## 4 Discussion

OFCs are one of the most common craniofacial birth defects in the world, posing a severe burden on infants, family and society. In the present research we reported the prevalence, death, DALYs, and their temporal trends over a 31-year period from 1990–2021 based on the latest data extracted from GBD 2021 at the global, regional, and national levels. Globally, the ASPR, ASMR, and ASDR of OFCs decreased from 1990–2021, demonstrated effectiveness of current preventive measures. However, the disease burden is unevenly distributed worldwide; some regions and countries continue to face increasing burdens, as evidenced by rising ASPR in Southeast Asia, the Caribbean, and Oceania, while ASMR remains on an upward trajectory in Afghanistan, Trinidad and Tobago, and Uzbekistan. In this study, the SDI index indicates a country's position on the development spectrum, which considers education, fertility, and poverty levels. The burden of OFCs varies with the SDI level; our findings reveal that 81.0% of the prevalence and 96.1% of deaths occurred in low- and middle-SDI countries. This is similar to the results of previous studies. Regions with high SDI exhibited the lowest burdens and the most significant decreasing trends in prevalence. Recent studies have also indicated that lower socioeconomic status correlates with a higher incidence of OFCs (18). Children with OFCs may have severe speech disorders, hearing loss, scar repair, nutrition, and mental and social developmental disorders. Therefore, OFCs needs to be solved through multidisciplinary sequential therapy starting from the neonatal period and running through the entire growth and development stage of patients. This requires the collaboration of orthopedic surgeons, plastic surgeons, dentists, pediatricians, otolaryngologists, physical therapists, etc., to gradually repair anatomical defects, restore function, and improve appearance (19). Areas with high SDIs are equipped with high-quality medical resources and team that provide targeted, sequential, and effective treatment for OFCs patients, compared to areas with a low SDI, therefore, they experience lower burdens.

The estimated overall global birth prevalence of 0.45 per 1,000 live births was calculated from a recent meta-data analysis (5). Our study estimated a global ASPR of OFCs at 53.42 cases per 100,000 population, higher than the previously published data. However, the prevalence of OFCs conditions varies across geographic areas and ethnic groupings. The low prevalence of OFCs in previous studies may be due to lack of information in areas with a higher prevalence, inclusion of countries or incomplete data (5)Previous reports of birth prevalence of OFCs vary considerably from different African populations. The prevalence of OFC is low in Africa with 0.5/1,000 cases in Nigeria (20), 0.44/1,000 cases in Ethiopia (21), and and 0.31/1,000 cases in South Africa (22). I In our research, sub-Saharan Africa had the lowest prevalence, with1.03/1,000 cases.

A review of the prevalence of OFCs in Africa and the Middle East showed an average prevalence of 1.25/1,000 live births (23). The prevalence of OFCs in the regions of Saudi Arabia ranges from 0.65–1.9/1,000 live births (24). Parental consanguineous marriage was the highest prevalent risk factor for OFCs cases in the included studies (24). In our study, the ASPR of OFCs in North Africa and the Middle East was 85.22 per 10,000 population. The prevalence of births of OFCs in Africa is lower than in other parts of the world. It is possible that inadequate monitoring systems and research programs for OFCs have undermined the accuracy of birth rate estimates in low- and middle-income countries.

Previous literature has indicated that drugs and air pollution, maternal tobacco smoking, and environmental tobacco smoke exposure, along with prenatal alcohol exposure, can increase the risk of OFCs at birth (2, 9, 10, 25, 26). The highest prevalence of OFCs has been reported in South and South-East Asia, with Pakistan having an OFC prevalence of 1.91/1000 (27)., 1.94/1,000 population in the Philippines (28), 1.90/1,000 population in Japan (22, 29), 1.4/1,000 livebirths in China (30), 0.73/1,000 births in India, and 1.64/1,000 live birth in Nepal (31). In our study, the ASPR of OFCs was notably higher in South Asia, with 109.09 (88.36–135.28) cases per 100,000 populations, and in East Asia, with 53.06 (43.39–64.75) cases per 100,000 populations. Moreover, A meta-analysis showed that smoking is an important risk factor for OFCs, and that smoking is significantly more strongly associated with the risk of OFCs in Asia and South America than in Europe and North America. Therefore, stringent restrictions on smoking are imperative in Asia (32).

The mortality rate of OFCs significantly decreased across most countries from 1990–2021. The primary cause of death is the syndromic type of OFCs (33). This notable reduction in mortality primarily resulted from the widespread adoption of universal prenatal diagnosis; earlier diagnosis and subsequent termination of foetuses with OFCs lessened the adverse impacts of pregnancy termination on the mother and her family (34). At present, many OFCs can be detected by B-ultrasonography, a universal and effective prenatal detection and diagnosis of congenital anomalies early in pregnancy, advanced ultrasonography (such as 3D/4D imaging) at 11 and 13 + 6 weeks of gestation can detect OFCs early, with a sensitivity of 90%–100% (35). Consequently, most mothers terminate their pregnancy as soon as possible after diagnosed with nonsyndromic OFCs. If the family decides to terminate the pregnancy, it is recommended to complete the pregnancy before 24 weeks to reduce the risk of surgery and ethical controversy. Nevertheless, in some countries, death rates are rising because of religious beliefs prohibiting abortion or poor economic conditions (36). As selective termination of pregnancy is sensitive and controversial, parents can still benefit from prenatal screening to prepare in advance for the birth of a child with orofacial clefts. In our study, the highest number of deaths cases in 2021 was also found in Western Sub-Saharan Africa, North Africa and the Middle East, and Eastern Sub-Saharan Africa.

We used DALYs to measure the burden of disease for the OFCs from 1990–2021 in 204 countries and territories. In 2021, at least 408,775 (95% UI: 252,320–671,120) OFCs per 100,000 population globally. The GBD data likely underestimates the global burden of disease since it fails to take into consideration other diseases related to OFCs, such as dental caries, facial dysmorphism, and malocclusion. We found that the high SDI region showed the biggest downward trend and the least ASRs of prevalence, as well as DALYs. Moreover, an negative linear relationship inverse relationship was noted between SDI values and both ASMR and ASDR in 21 geographic regions. Recent studies have also indicated that lower socioeconomic status correlates with a higher incidence of OFCs (18, 36, 37). Lower SDI may be associated with nutritional deficiencies, inadequate prenatal folic acid intake, limited access to health care, insufficient health infrastructure, inadequate levels of education, and lack of knowledge and awareness of environmental risk factors that can lead to adverse pregnancy outcomes (18, 38).Although the prevalence of OFCs is high in some high SDI regions, such as the Republic of Korea, Japan, Finland, Switzerland, and Germany, the ASMR and ASDR of the disease remains low. Thus, a high level of treatment of OFCs is associated with a low burden of disease.

The major barriers to care for OFCs in patients from low- and middle-income countries were: patient travel costs, lack of patient awareness, and insufficient financial support. Patient travel costs emerged as the most frequently reported barrier in sub-Saharan Africa, the Middle East and North Africa, and South and Southeast Asia. In a survey of 68 multidisciplinary practitioners of OFCs care in 17 countries in Africa, “Patient travel costs” was the most commonly reported barrier to OFCs care (39). Patient travel costs, also represents one of the largest out-of-pocket expenditures encountered when seeking healthcare. Patients may need to travel long distances to receive care and poor infrastructure may make in-country travel prohibitively expensive. They often struggle to afford additional nonmedical costs like travel, accommodation and meals. In contrast, inadequate financial support is the main barrier in the Eastern Europe and East Asia (39). In low- and middle-income areas of sub-Saharan Africa, where infrastructure is often inadequate, international assistance is important for controlling OFCs (40). International mission assistance organisations should focus on enhancing local medical capacity to ensure sustainability (41). Effective collaborations between international host countries' assistance and visiting organisations are crucial in addressing the complex challenges associated with OFCs care in these regions (42). Additionally, strengthening censuses at the national level to establish accurate health data on OFCs and dynamic monitoring of the burden can help further OFCs prevention and treatment. In addition, there is limited search for medical information on OFCs, so there is a need to utilize social media to provide educational resources for families with OFCs (4). Therefore, enhanced nutritional intake, emphasis on health care worker training, improved public awareness of preventive health care and allocation of health care resources in low-income countries may effectively reduce the disease burden of OFCs.

Our study has few limitations. First, this research is a secondary analysis of the GBD study data, and the accuracy and reliability of the estimates are still insufficient. Many low - and middle-income countries, particularly in Africa, South Asia and elsewhere, have inadequate disease surveillance systems, variations in healthcare reporting systems, missing or incomplete data, the registration of orofacial clefts may be missed in the low economic regions, leading to an underestimation of the disease burden. Second, in the GBD 2021 database, cleft lip and cleft palate are combined as OFCs, and in this study we were unable to distinguish whether it was unilateral or bilateral cleft lip or cleft palate, and whether there was a combined cleft. It is hoped that more detailed data on the types of cleft lip will be collected in the future to better understand the epidemiological characteristics of different types of cleft lip and palate. Thirdly, the risk factors are not sufficiently identified to explain the regional differences and temporal patterns in the disease burden of orofacial clefts. More data on risk factors are needed for further research.

## 5 Conclusions

OFCs represent a significant public health challenge globally, yet the geographical distribution of this burden varies. The global burden of OFCs decreased from 1990–2021. However, some countries continuing to experience increasing burdens. The greatest burden of OFCs was noted in South Asia, North Africa, the Middle East, and East Asia, where over 80% of the burden is borne by patients in low- and middle-income countries. Enhanced public awareness of OFCs prevention, stringent restrictions on smoking, control of air pollution, improved healthcare infrastructure, and increased training for healthcare workers in low- and middle-income countries are essential to mitigate the future burden of this disease.

## Data Availability

The original contributions presented in the study are included in the article/Supplementary Material, further inquiries can be directed to the corresponding author.
