# Supplementary material for: Burden of orofacial clefts from 1990–2021 at global, regional, and national levels
Source: Front Pediatr. 2025 Mar 21;13:1502877. doi: 10.3389/fped.2025.1502877 (PMC11968431; doi:10.3389/fped.2025.1502877)
Supplement: Supplementary file 1 [file Datasheet1.pdf]

Additional file 1

Burden of orofacial clefts from 1990 to 2021 at global, regional, and national levels

Fig S1: The prevalance cases of orofacial clefts in 2021 for 21 GBD regions, by sex……………………………………………... ……………. ……….………….2

Fig S2: The age-standardized death rate orofacial clefts in 2021 for 21 GBD regions, by sex……………………………………………………………………...….3

Fig S3: The death cases of orofacial clefts in 2021 for 21 GBD regions, by sex………….. ……………….. ………………….……………………………………… 4

Fig S4: The DALYS cases of orofacial clefts in 2021 for 21 GBD regions, by sex………………………………………………………………………………….……….5

Fig S5: The death cases of orofacial clefts in 2021 for 204 countries and territories ……………… ……………………………………………………………………6

Fig S6: The ASMR of orofacial clefts in 2021 for 204 countries and territories ………………………. ………………………………………………………………7

Fig S7: The ASMR chage of orofacial clefts in 2021 for 204 countries and territories …………………………………. …………………..…. ………….……..8

Fig S8: The ASDR of orofacial clefts in 2021 for 204 countries and territories …………………………..………………………………………………………. 9

Fig S9: The ASDR chage of orofacial clefts in 2021 for 204 countries and territories ………………………………………. …………………………..……..   10

Fig S10: The DALYs cases of orofacial clefts in 2021 for 204 countries and territories ……………………..………. …….….……………………….……. 11

Fig S11: Global and 21 regions both sexes   age-standardized prevalence rate 1990-2021 with SDI.…………………………………. ……………….…. 12

Fig S12: Global and 21 regions both sexes deaths age-standardized rate 1990-2021 with SDI …………………………………………………………………. 13

Fig S13: Age-standardized prevalence rates for orofacial clefts for 204 countries and territories by SDI, 2021. …………………………………….. 14

Fig S14: Age-standardized deaths rates for orofacial clefts for 204 countries and territories by SDI, 2021. ……………………………………………15

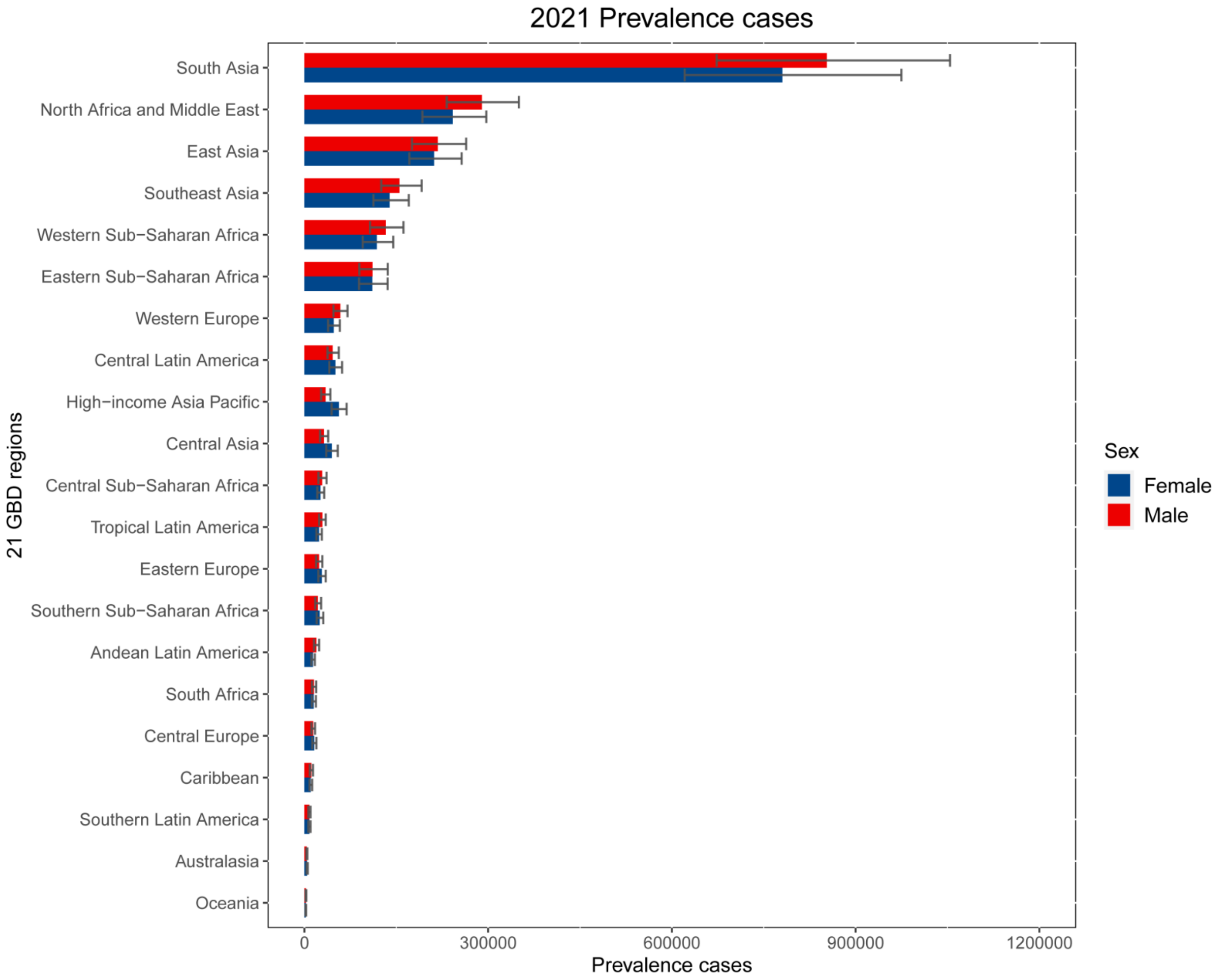

Fig S1: The prevalance cases of orofacial clefts in 2021 for 21 GBD regions, by sex

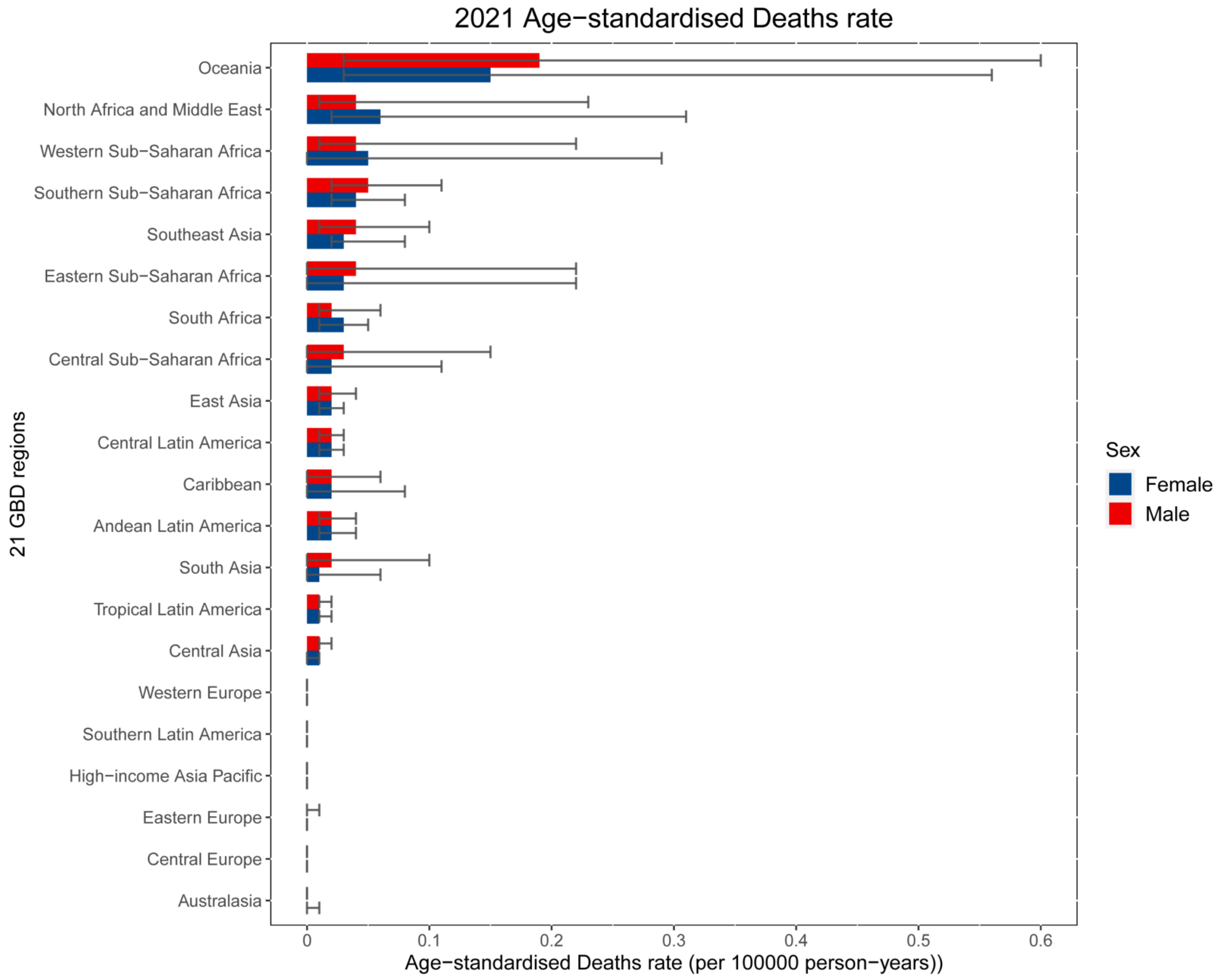

Fig S2: The age-standardized death rate orofacial clefts in 2021 for 21 GBD regions, by sex

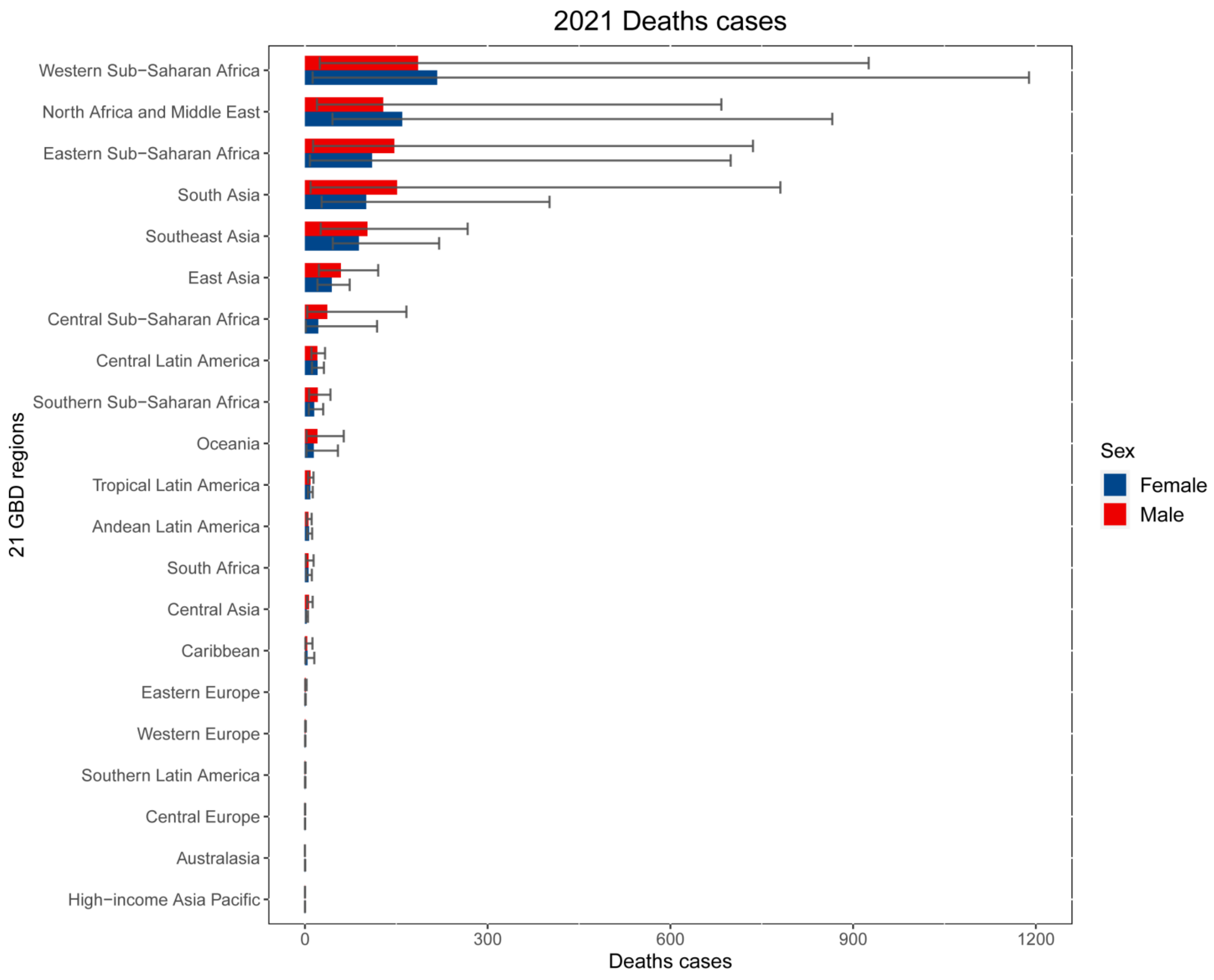

Fig S3: The death cases of orofacial clefts in 2021 for 21 GBD regions, by sex

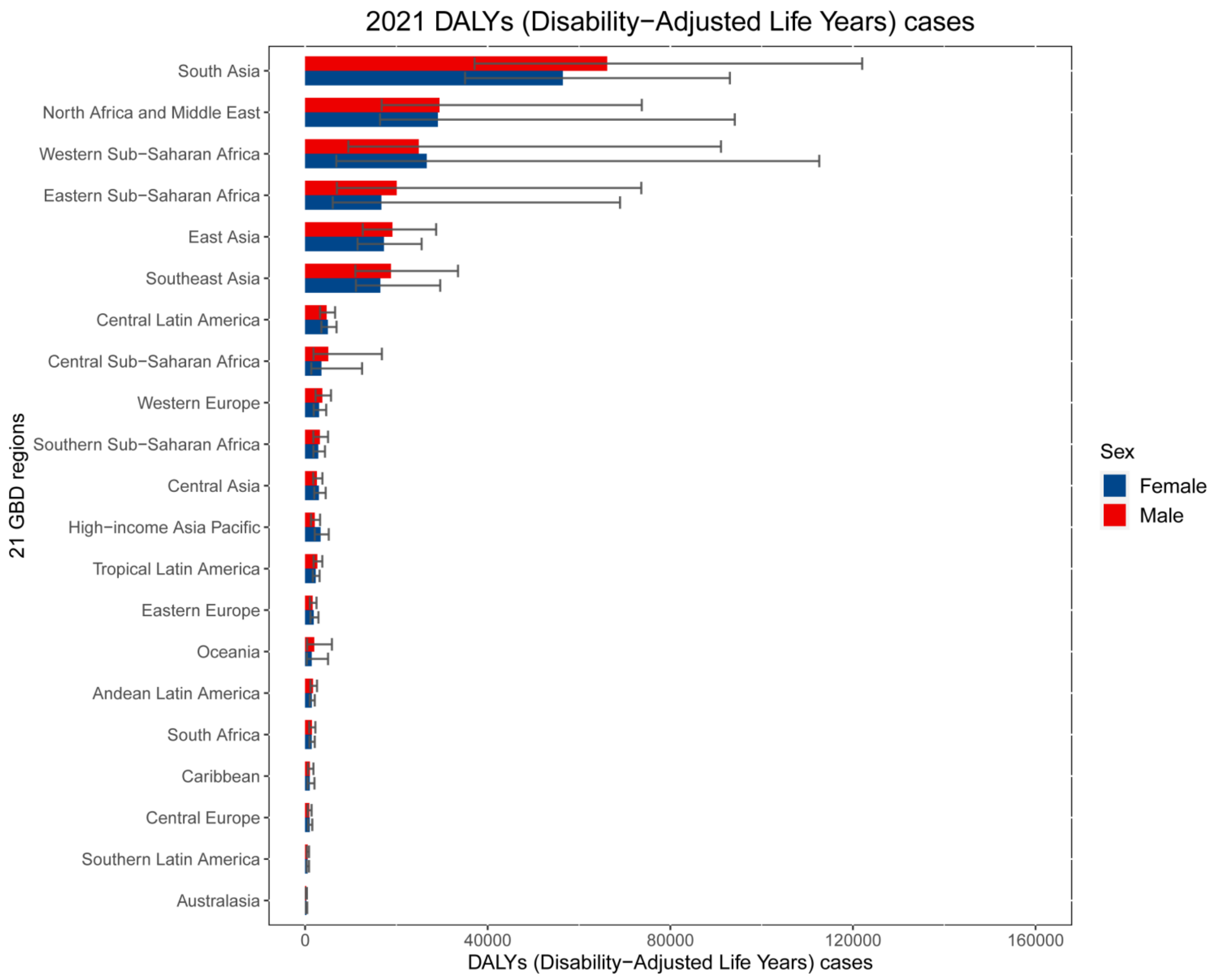

Fig S4: The DALYS cases of orofacial clefts in 2021 for 21 GBD regions, by sex

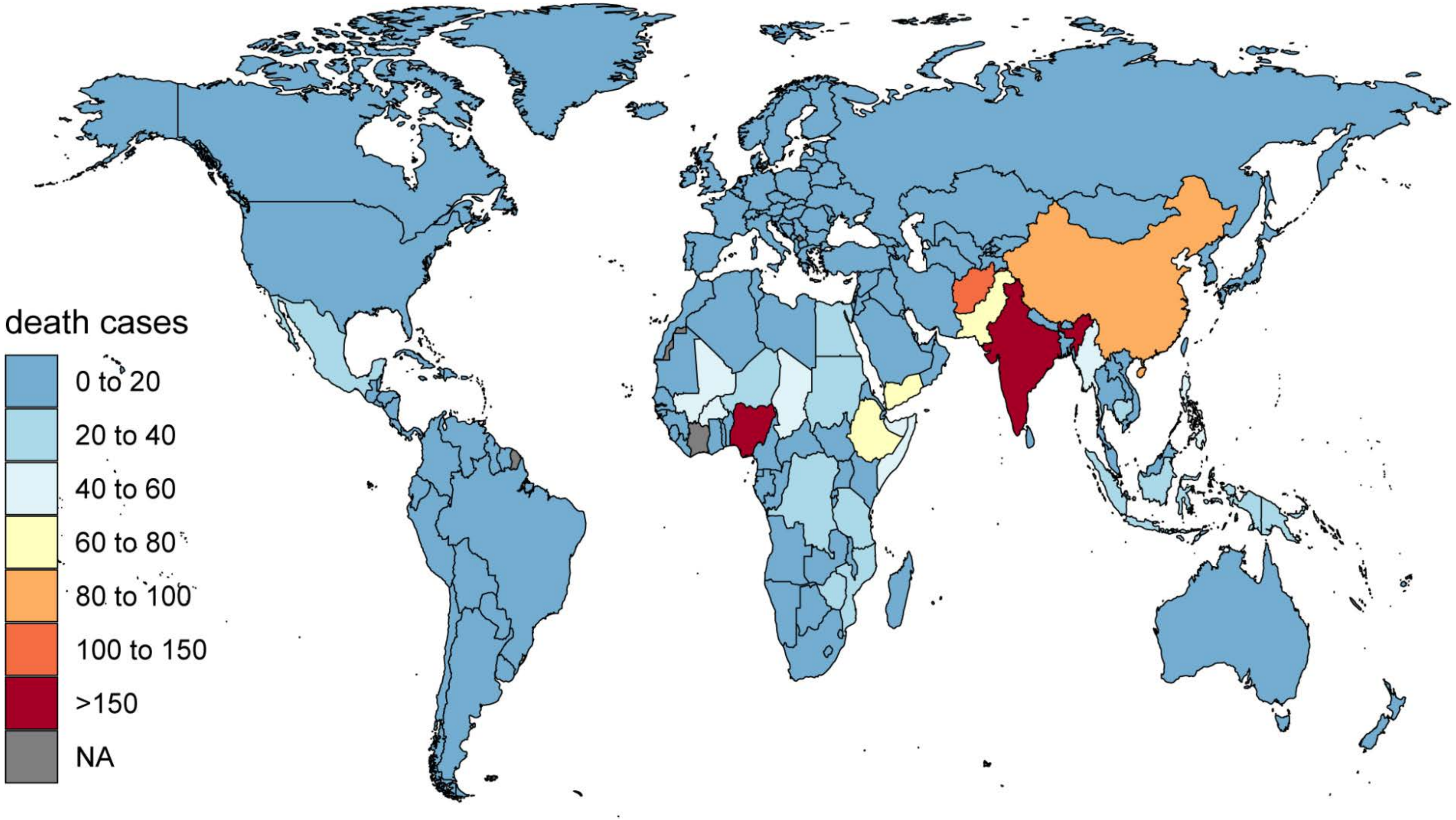

Fig S5: The death cases of orofacial clefts in 2021 for 204 countries and territories

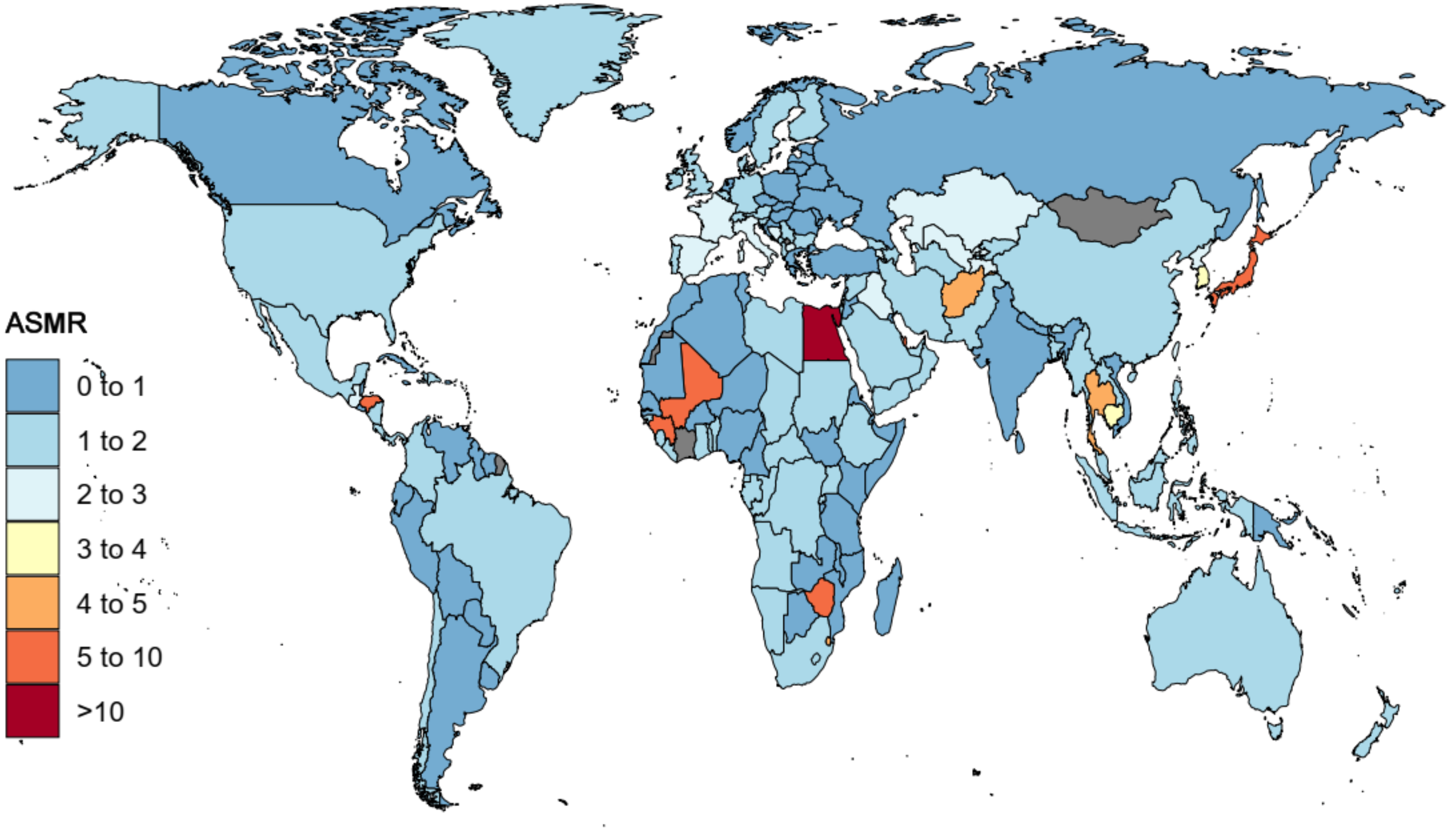

Fig S6: The ASMR of orofacial clefts in 2021 for 204 countries and territories

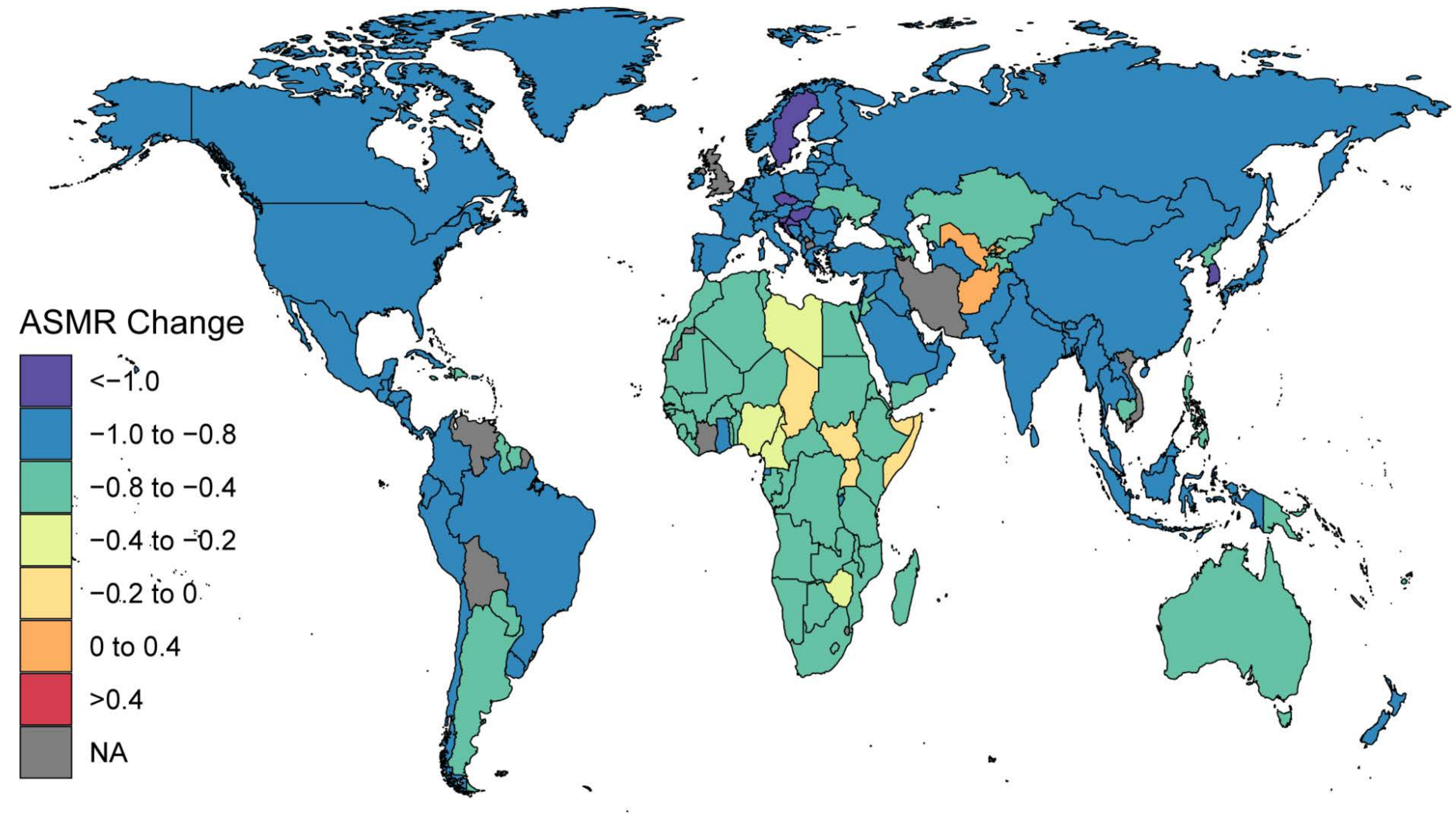

Fig S7: The ASMR chage of orofacial clefts in 2021 for 204 countries and territories

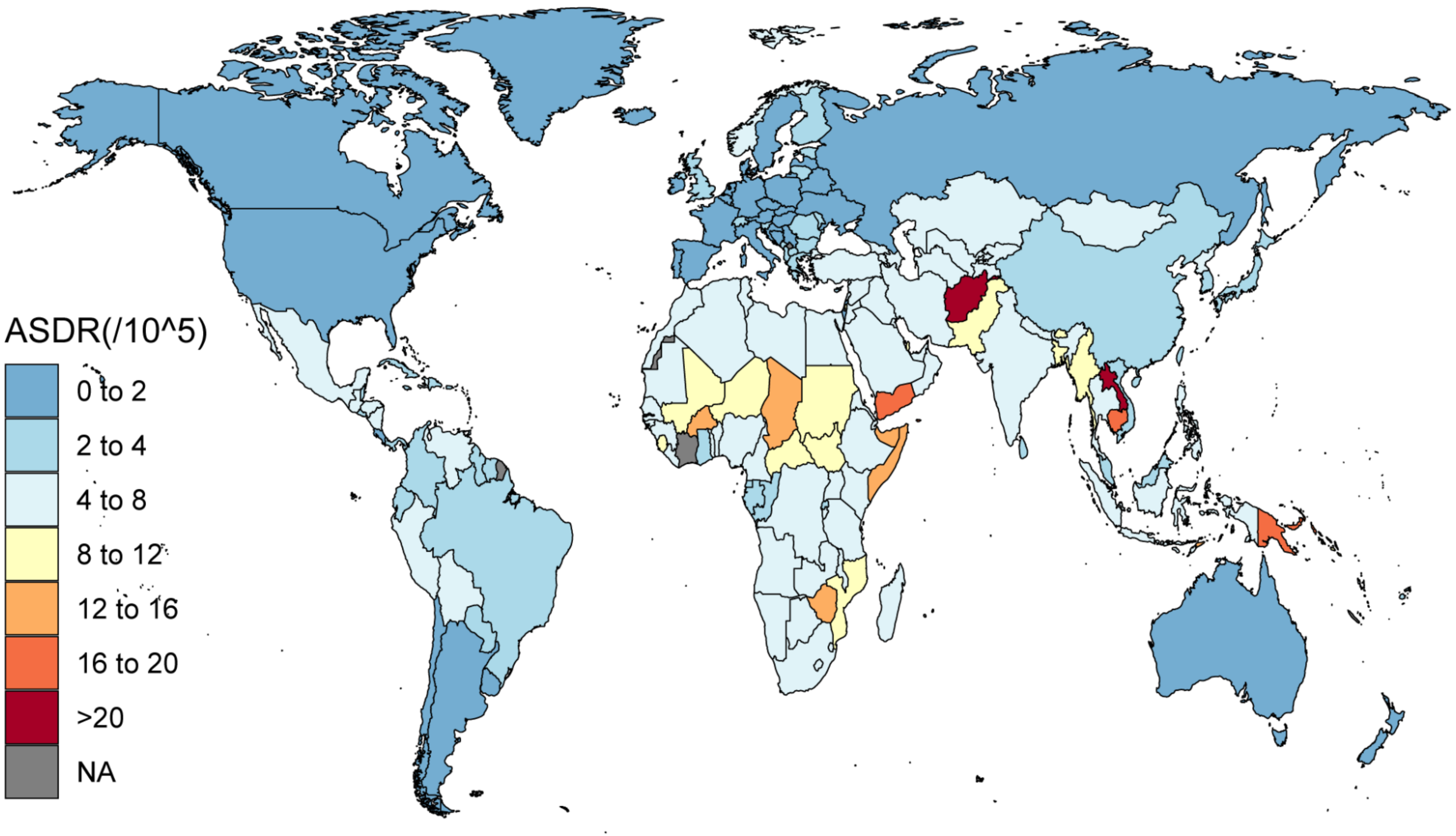

Fig S8: The ASDR of orofacial clefts in 2021 for 204 countries and territories

.

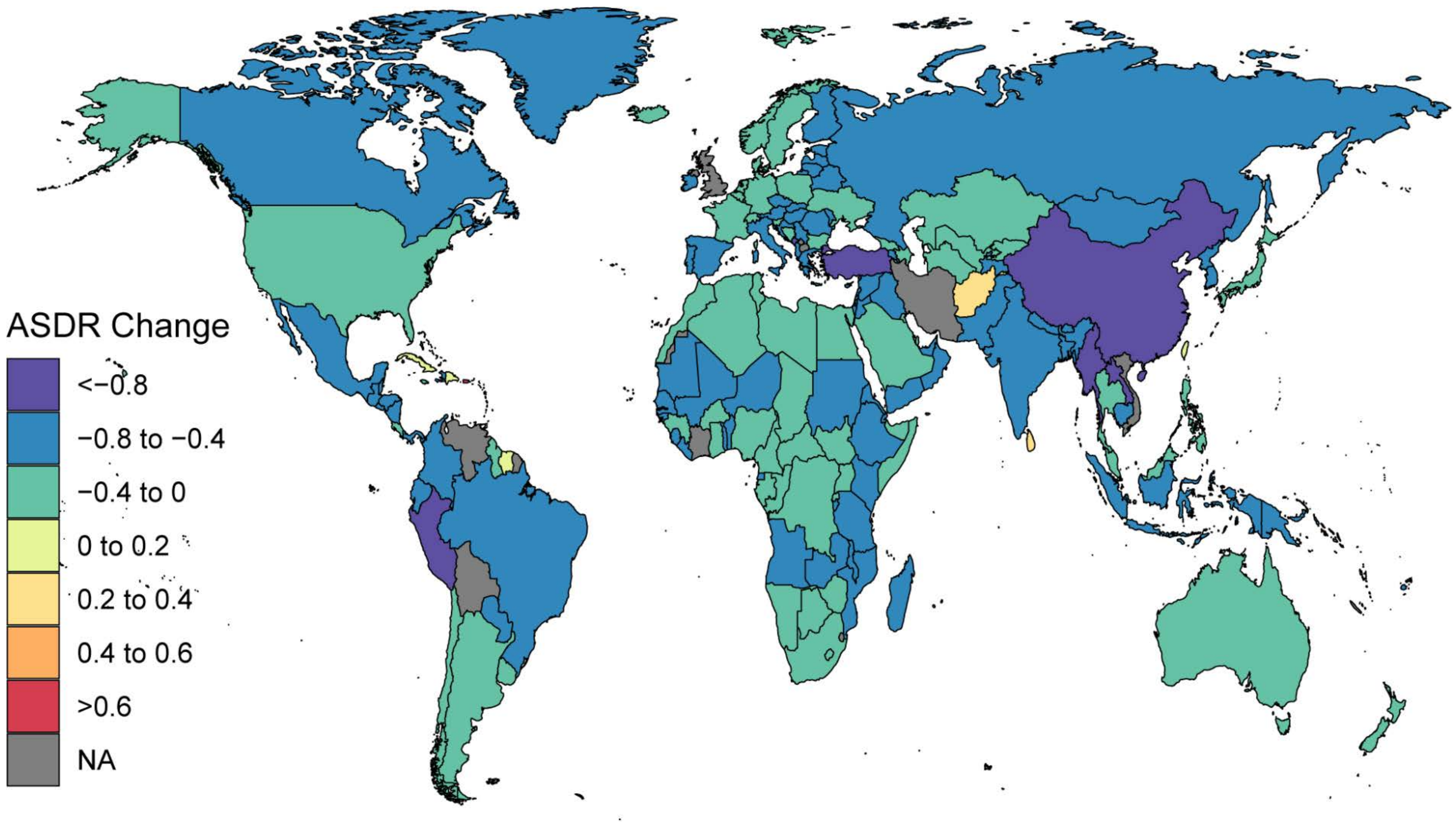

Fig S9: The ASDR chage of orofacial clefts in 2021 for 204 countries and territories

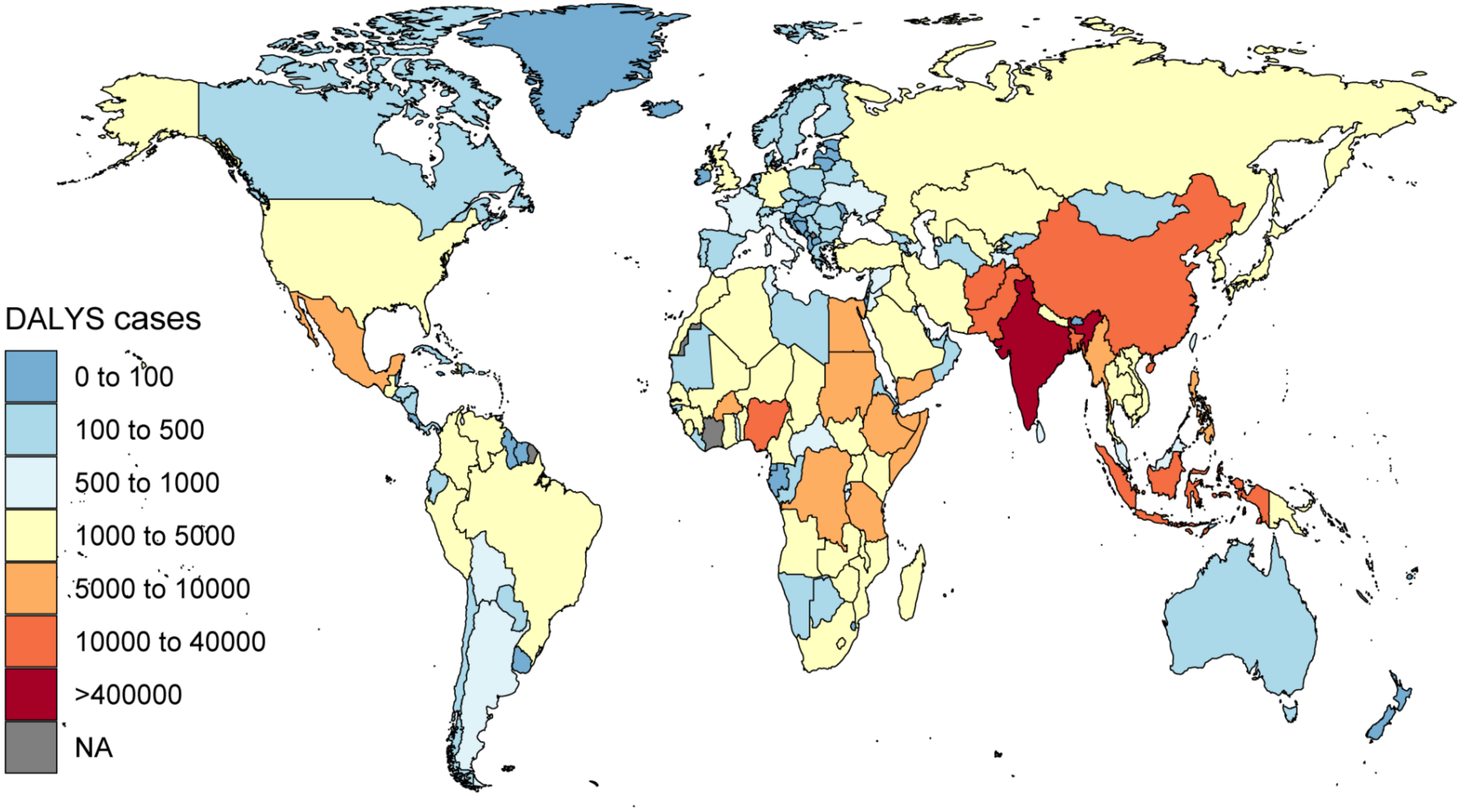

Fig S10: The DALYs cases of orofacial clefts in 2021 for 204 countries and territories

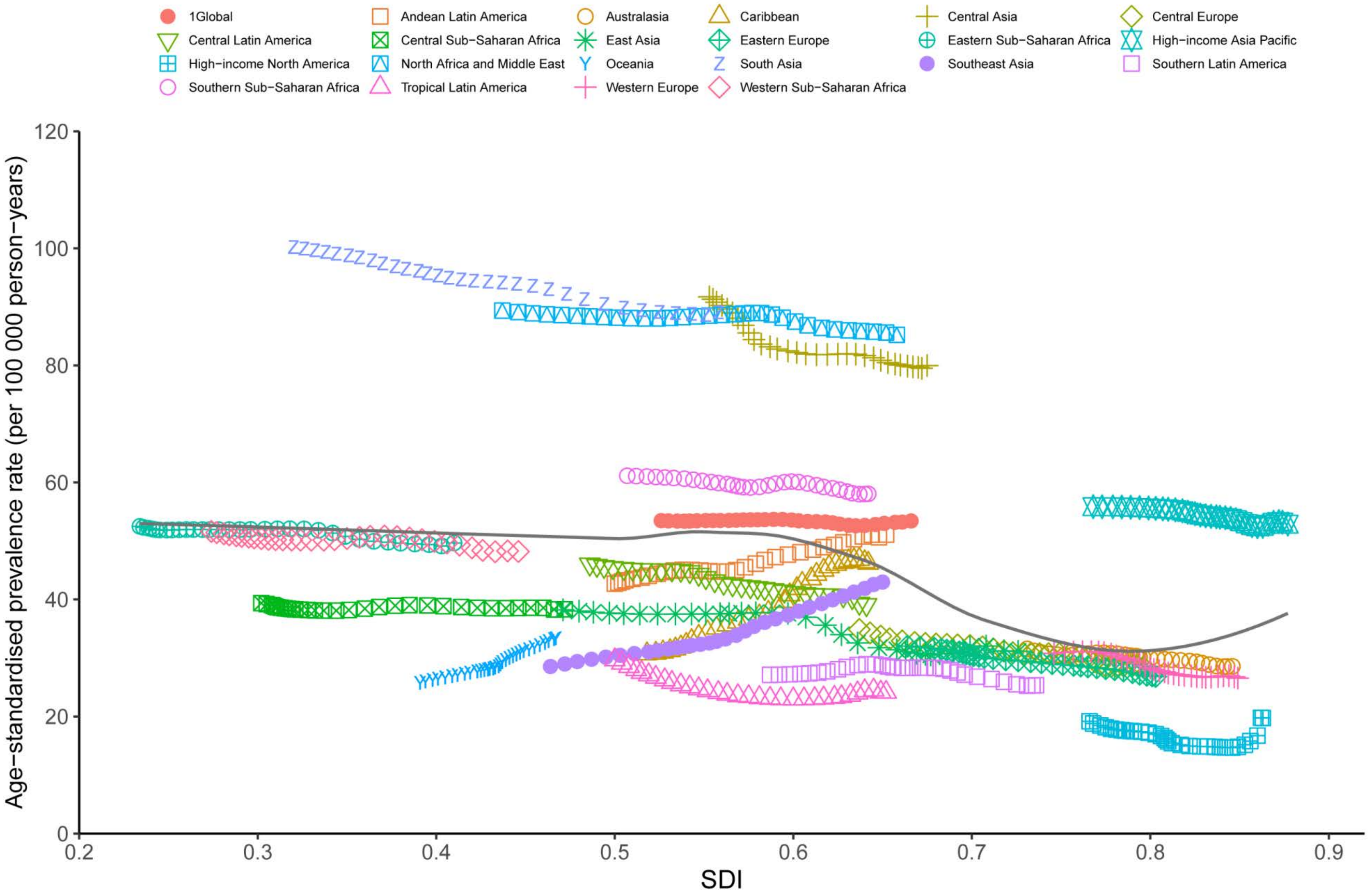

Fig S11: Global and 21 regions both sexes age-standardized prevalence rate 1990-2021 with SDI

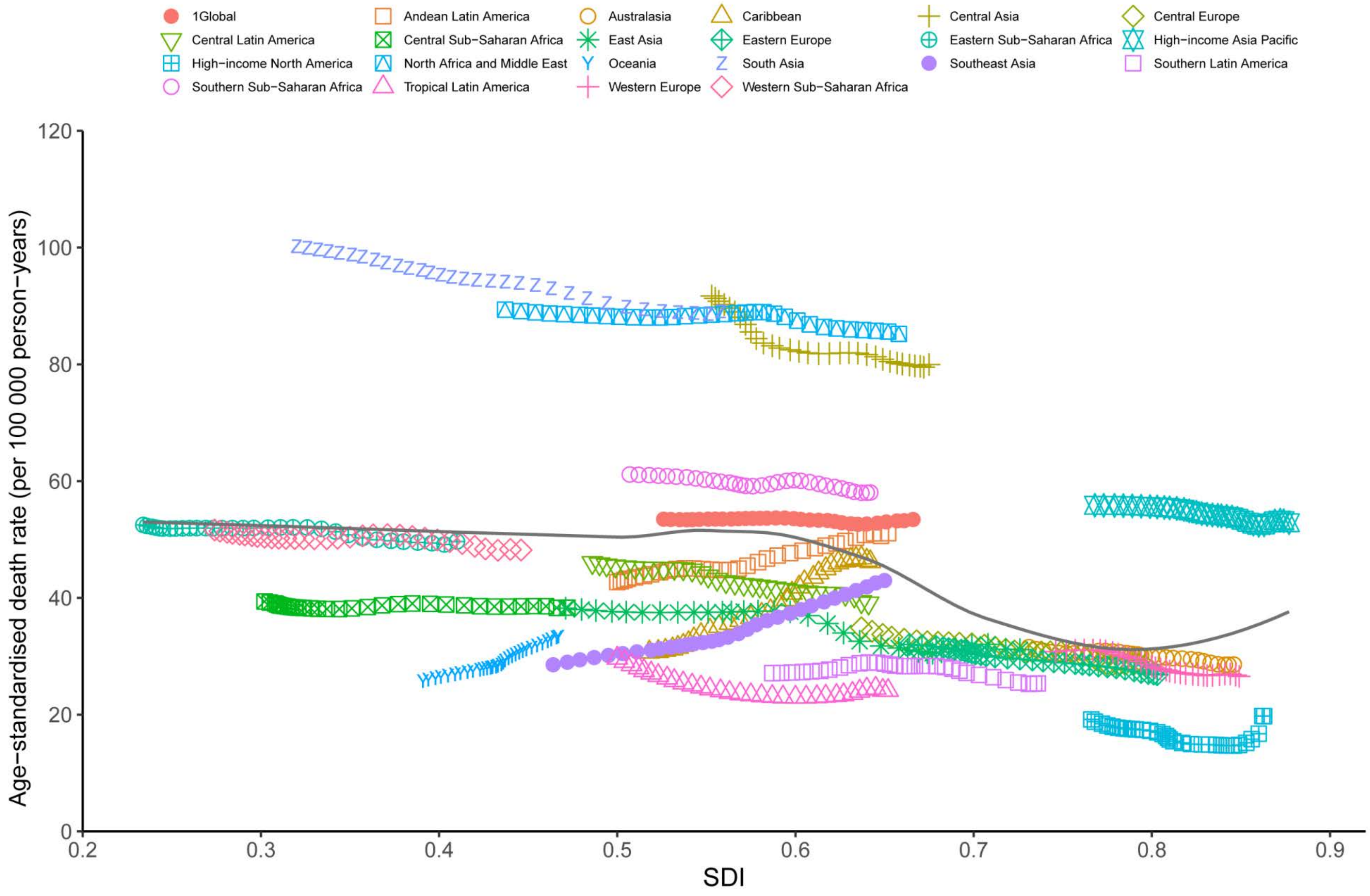

Fig S12: Global and 21 regions both sexes deaths age-standardized rate 1990-2021 with SDI.

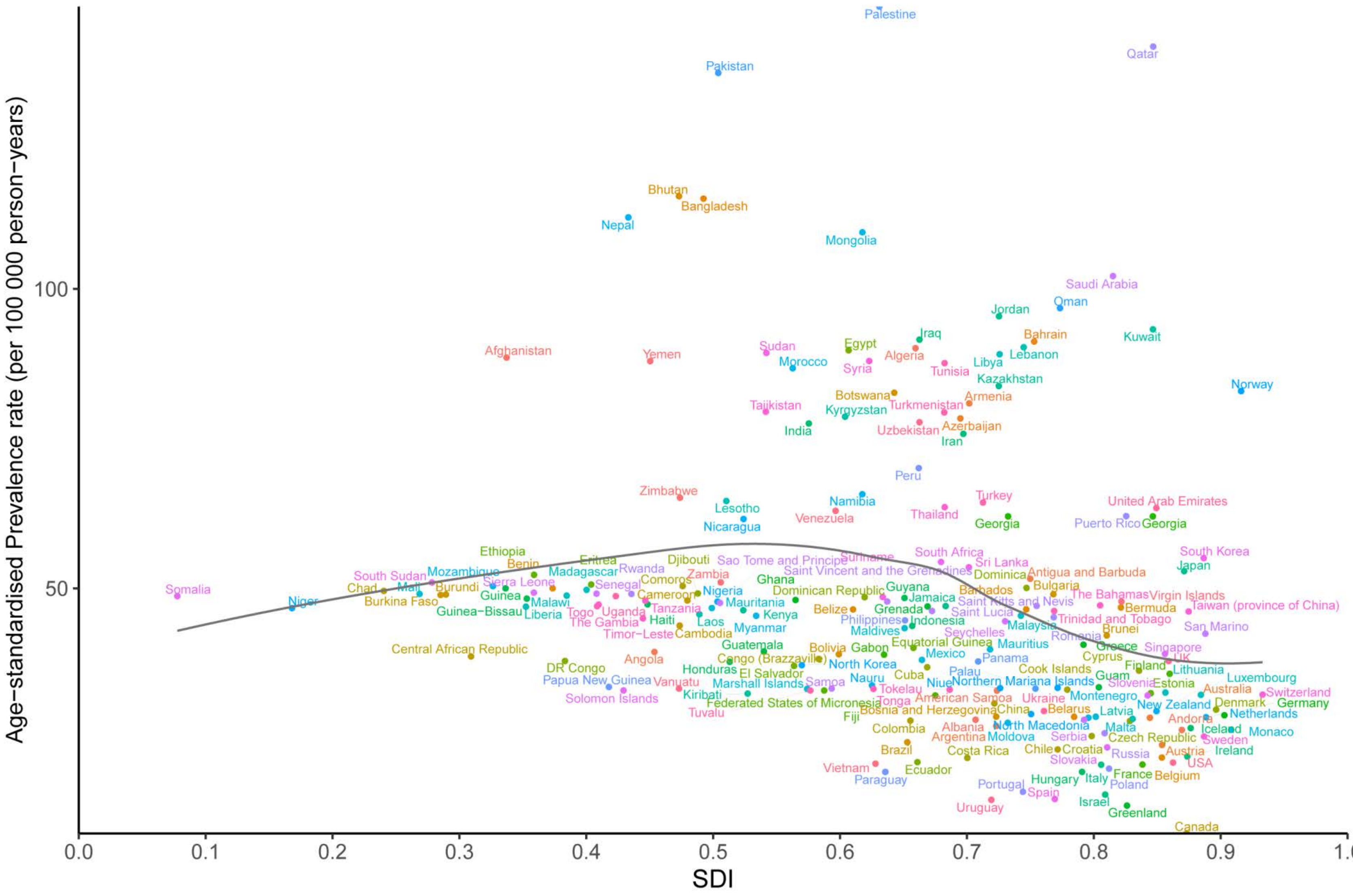

Fig S13: Age-standardized prevalence rates for orofacial clefts for 204 countries and territories by SDI, 2021.

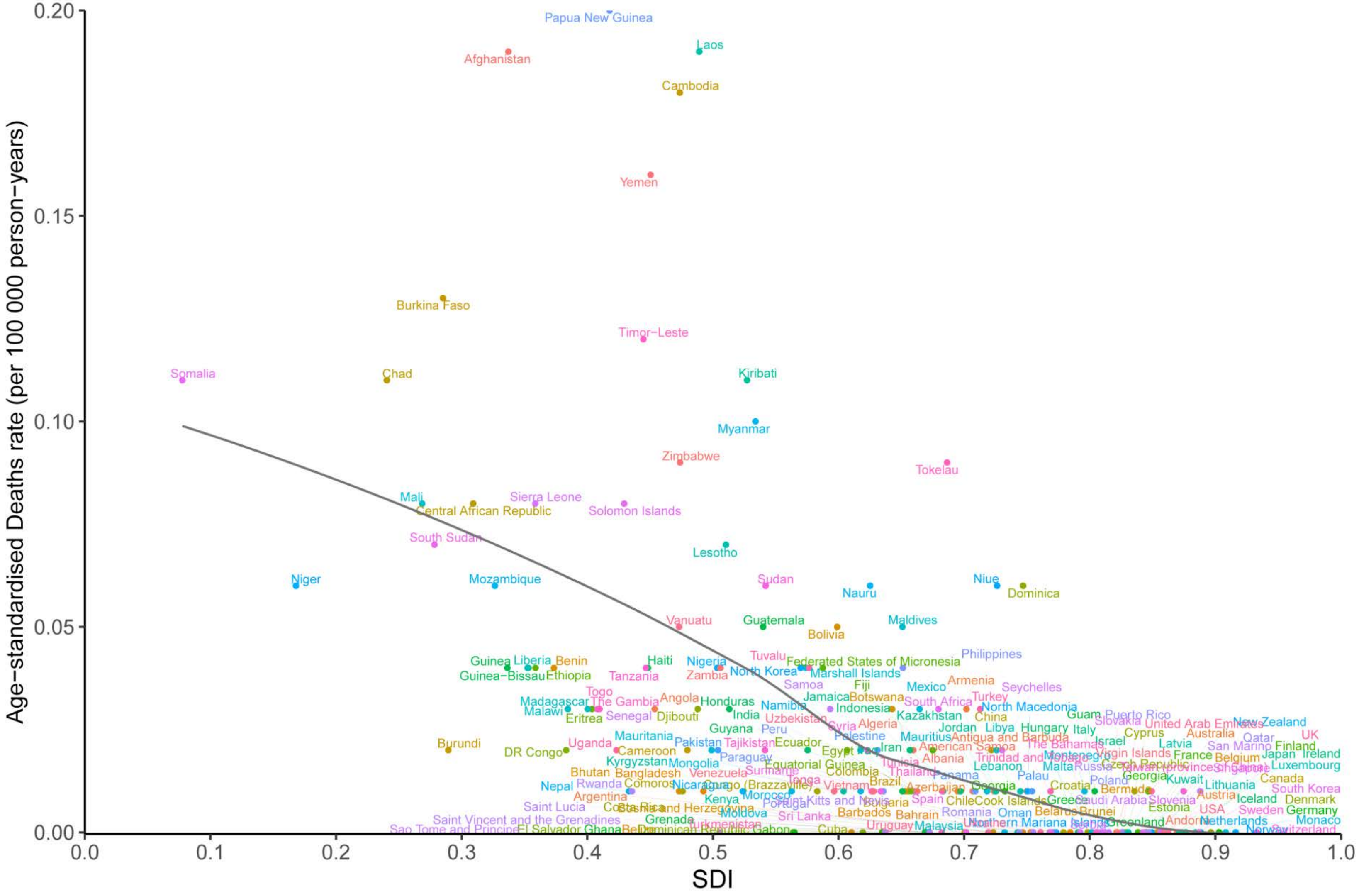

Fig S14: Age-standardized deaths rates for orofacial clefts for 204 countries and territories by SDI, 2021.
